# Diversity and intratumoral heterogeneity in human gallbladder cancer progression revealed by single‐cell RNA sequencing

**DOI:** 10.1002/ctm2.462

**Published:** 2021-06-27

**Authors:** Peizhan Chen, Yueqi Wang, Jingquan Li, Xiaobo Bo, Jie Wang, Lingxi Nan, Changcheng Wang, Qian Ba, Houbao Liu, Hui Wang

**Affiliations:** ^1^ State Key Laboratory of Oncogenes and Related Genes Center for Single‐Cell Omics School of Public Health Shanghai Jiao Tong University School of Medicine Shanghai China; ^2^ Department of General Surgery Zhongshan Hospital Fudan University Shanghai China; ^3^ Biliary Tract Diseases Institute Fudan University Shanghai China; ^4^ Cancer Center Zhongshan Hospital Fudan University Shanghai China

**Keywords:** gallbladder cancer, intercellular communications, myeloid cells, scRNA‐seq, T cells, trajectory analysis, tumor microenvironment

## Abstract

**Background:**

Gallbladder cancer (GC) is a malignant disease characterized with highly cellular heterogeneity and poor prognosis. Determining the intratumoral heterogeneity and microenvironment (TME) can provide novel therapeutic strategies for GC.

**Methods:**

We performed the single‐cell RNA sequencing on the primary and lymph node metastatic gallbladder tumors and the adjacent normal tissues of five patients. The transcriptomic atlas and ligand–receptor‐based intercellular communication networks of the single cells were characterized.

**Results:**

The transcriptomic landscape of 24,887 single cells was obtained and characterized as 10 cellular clusters, including epithelial, neuroendocrine tumor cells, T&NK cells, B cells, RGS5+ fibroblasts, POSTN+ fibroblasts, PDGFRA+ fibroblasts, endothelial, myeloid cells, and mast cells. Different types of GC harbored distinct epithelial tumor subpopulations, and squamous cell carcinoma could be differentiated from adenocarcinoma cells. Abundant immune cells infiltrated into adenocarcinoma and squamous cell carcinoma, rather than neuroendocrine neoplasms, which showed significant enrichment of stromal cells. CD4+/FOXP3+ T‐reg and CD4+/CXCL13+ T helper cells with higher exhausting biomarkers, as well as a dynamic lineage transition of tumor‐associated macrophages from CCL20^hi^/CD163^lo^, CCL20^lo^/CD163^hi^ to APOE+, were identified in GC tissues, suggesting the immunosuppressive and tumor‐promoting status of immune cells in TME. Two distinct endothelial cells (KDR+ and ACKR1+), which were involved in angiogenesis and lymphangiogenesis, showed remarkable ligand–receptor interactions with primary GC cells and macrophages in gallbladder tumors.

**Conclusions:**

This study reveals a widespread reprogramming across multiple cell populations in GC progression, dissects the cellular heterogeneity and interactions in gallbladder TME, and provides potential therapeutic targets for GC.

## Graphical abstract

- Abundant immune cells in adenocarcinoma and squamous cell carcinoma while stromal cells in neuroendocrine neoplasms were enriched.
- Immunosuppressive microenvironment characterized as exhausted T cells and APOE+ macrophages.
- Endothelial cells (KDR+ and ACKR1+) indicated that angiogenesis and lymphangiogenesis were involved in GC.
- There were remarkable ligand–receptor interactions between endothelial, primary GC cells, and macrophages.

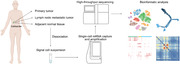

**Abbreviations**
CNVs: copy number variations
EMT: epithelial–mesenchymal transition.
GC: gallbladder cancer
NET: neuroendocrinology tumor
scRNA‐seq: single‐cell RNA sequencing
TILs: tumor‐infiltrating lymphocytes
TME: tumor microenvironment
UMI: unique molecular identifier

## 1 INTRODUCTION

Gallbladder cancer (GC), as a relatively rare malignancy, is usually originated from the epithelial cells in the biliary duct system. The prognosis of GC is poor with the median survival time less than 1 year and the 5‐year overall survival (OS) rate less than 5% according to the Surveillance, Epidemiology, and End Results program database.^1^ Curative surgery treatment is the most effective approach; however, only less than 10% of the patients are eligible for the surgery treatment at diagnosis due to the asymptomatic characteristics at the early stage, insidious onset, and rapid progression of the disease.^2^ Although chemotherapy, targeted therapy, and immune approaches provide other treatment choices, only a few patients yield promising prognoses. More potential therapeutic targets need to be explored for the effective treatment of this disease.

Previously genomic and transcriptomic studies based on next‐generation sequencing and microarray‐based methods have identified hotspot mutations or aberrantly expressed genes in signaling pathways that may lead to gallbladder tumorigenesis and progression.^3^, ^4^, ^5^, ^6^ Somatic mutations on TP53, KRAS, ERBB3, PIK3CA, and CTNNB1 were frequently noticed in gallbladder tumor samples, which may influence the clinical outcomes and the conventional chemotherapeutic treatment in the clinic.^5^, ^6^ However, these factors only account for a small proportion of GC patients and provide limited improvement for targeted therapy in the clinic. Meanwhile, these studies were performed in the bulk of tumors and the cellular heterogeneity in cancer tissues had not yet been well determined.^4^ Besides cancer cells, other cell types in the tumor microenvironment (TME) including stromal cells, tumor‐infiltrating lymphocytes (TILs), endothelial cells, and myeloid cells have been reported to be critical for the proliferation, metastasis, angiogenesis, immune evasion, and drug resistance of cancer cells.^7^ In particular, the properties of TILs are associated with the responses to immune checkpoint blockade treatments and the prognosis of patients.^8^, ^9^ However, the intercellular communications between TME cells and tumor cells and their roles in GC development and progression are still largely unknown.

Single‐cell RNA sequencing (scRNA‐seq) could provide valuable information regarding the cellular heterogeneity for cancer cells and the characteristics of distinct subpopulations in TME as it allows to massively determine the transcriptomes of thousands of cells at a time.^10^, ^11^, ^12^ In the current study, for the first time, we dissected the intratumoral heterogeneity and the TME characteristic cells in primary, lymph node metastatic tumor tissues as well as the adjacent normal bile duct tissues by scRNA‐seq. This study would provide deeper insights into the tumor heterogeneity of GC cells, determine the cellular characteristics of TME in GC patients, and improve our current understanding of the mechanisms of GC development and progression.

## 2 RESULTS

### 2.1 Single‐cell RNA expression profiling of human GC

**FIGURE 1:**
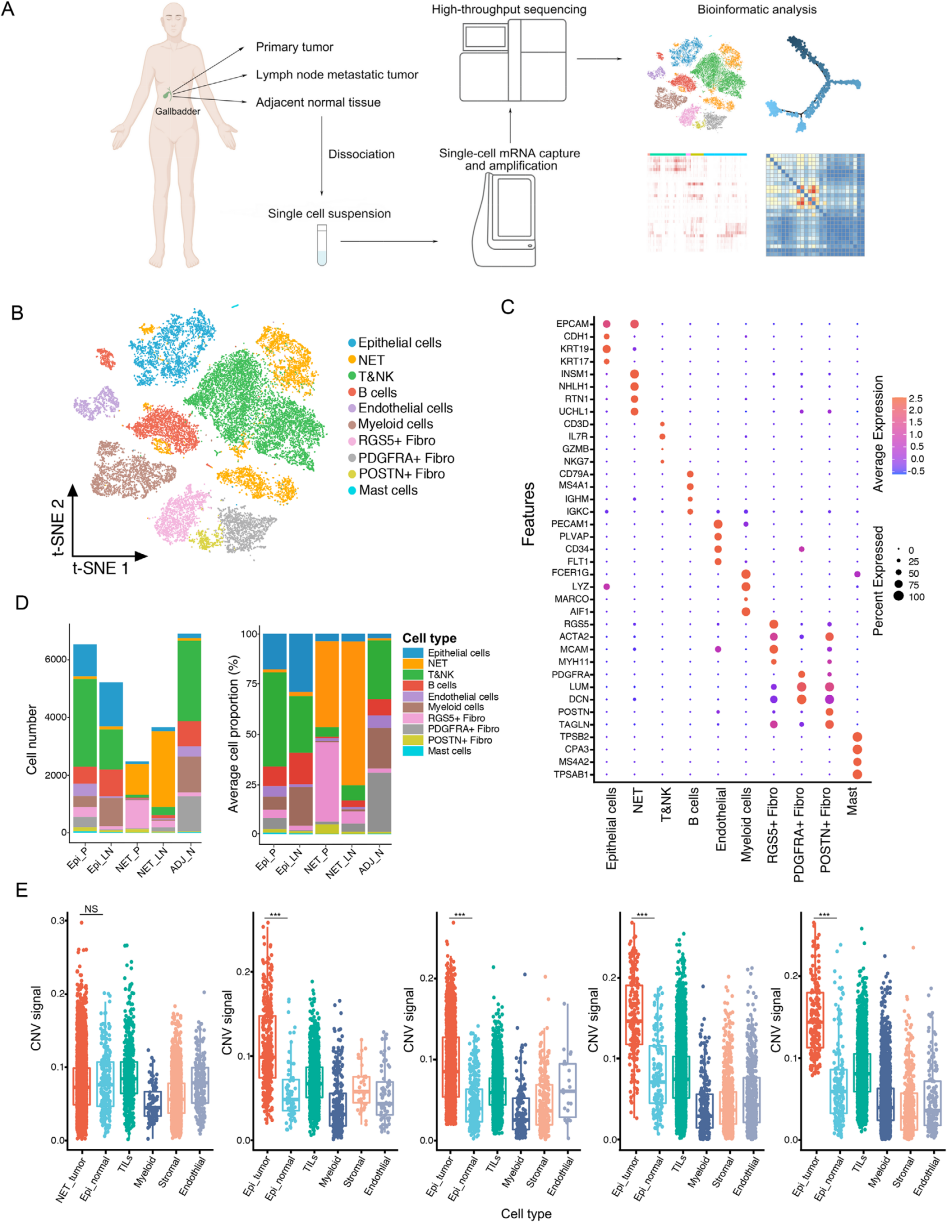
Distinct cell types in GC and the adjacent normal tissues identified through integrating single‐cell transcriptomic data. (A) Overview of the study design, sample collection, single cell preparation, sequencing, and bioinformatic analysis. (B) The t‐SNE plot identified 10 main cell types in GC and adjacent normal tissue. Resolution used for t‐SNE cell grouping analysis is 2.5. (C) The dot plot of the expression level of representative well‐known biomarkers across distinct cell types. The *x*‐axis indicated distinct cell subtypes and the *y*‐axis indicated specific biomarkers of each cell subgroup. (D) The number (left) and average proportion (right) of assigned cell types in different tissue types were presented. The color panel indicated different cell subgroups in the scRNA‐seq data (Epi_P, *n* = 4; Epi_LN, *n* = 3; NET_P, *n* = 1; NET_LN, *n* = 1; ADJ_N, *n* = 3). (E) Boxplot showing distributions of CNV scores among different cellular types in gallbladder tissues from patient SC128, SC110, SC133, SC144, and SC146, respectively. ^***^ *p* < 0.001 for Wilcoxon test (two‐tailed). NS, no significance. Epi_P, primary epithelial (adenocarcinoma or squamous) tumor tissue; Epi_LN, lymph node metastatic epithelial (adenocarcinoma or squamous) tumor tissue; NET_P, primary neuroendocrine tumor; NET_LN, lymph node metastatic neuroendocrine tumor; ADJ_N, adjacent normal tissue

To explore the cellular diversity and microenvironment composition in GC, we performed the scRNA‐seq analysis of primary GC tumors, lymph node metastatic tumors, and the adjacent normal tissues from three patients with gallbladder adenocarcinoma, one patient with adenosquamous cell carcinoma, and one patient with gallbladder neuroendocrine tumor (NET) (Table S1 and Figure S1A). After quality control assessment, we acquired transcriptomes from 24,887 single cells including 9040 cells from primary tumor tissues, 8917 cells from lymph node metastatic tumor tissues, and 6930 cells from adjacent normal tissues using the BD Rhapsody™ single‐cell mRNA whole transcriptome analysis system (Figure 1A). According to the t‐distributed Stochastic Neighbor Embedding (t‐SNE) analysis and canonical markers expression, 10 distinct cell populations were identified from the whole single‐cell profiling (Figures 1B and 1C), including the epithelial cells, NET cells, T&NK cells, B cells, endothelial cells, myeloid cells, RGS5+ fibroblasts, PSOTN+ fibroblasts, PDGFRA+ fibroblasts, and mast cells (Table S2). These cell populations distributed unevenly among patients or lesion sites (Figures 1D and S1B–S1E). The CDH1+/EPCAM+ epithelial cells were enriched in adenocarcinoma and adenosquamous tissues but rarely noticed in NET, in which the EPCAM+/INSM1+ neuroendocrine cells were exclusively identified (Figures 1D and S1C). The most abundant immune cells in epithelial GC tumors and the corresponding normal tissues included T&NK lymphocytes, B cells, and myeloid cells. In comparison, all these immune cells showed a lower proportion in the NET tissues (Figures 1D and S1C). Notably, the stromal cells including RGS5+ fibroblasts, PSOTN+ fibroblasts, and PDGFRA+ fibroblasts were predominantly enriched in primary and lymph node metastatic NET tissues (Figure 1D). The differentially expressed genes (DEGs) between these cellular subclusters were shown in Figure S1F and Table S2. These results unveil the tumoral and TME heterogeneity among different types of GC.

### 2.2 Heterogeneity of gallbladder epithelial tumor and NET cells

The gallbladder adenocarcinoma and adenosquamous cell carcinoma are originated from mucosa epithelial cells in the gallbladder, while the NET is a rare neoplasm that originates and spreads from the neuroendocrine cells or peptidergic neural crest Kulchitsky cells.^13^ The normal and cancerous epithelial cells resident in GC tissues could be distinguished through the genomic copy number variations (CNVs). Comparing with patient‐paired immune cells, stromal cells, and epithelial cells in normal tissues, the epithelial in tumor tissues showed markedly higher CNV score (Figure 1E), confirming the genomic variations and malignancy of epithelial cells in tumor tissues. However, no significant elevation of CNV score in NET cells compared to epithelial normal cells was observed (Figure 1E), suggesting that other genomic variations (e.g., gene mutations or epigenetic alterations) may play important roles in the malignant development and transformation of NET cells.

**FIGURE 2:**
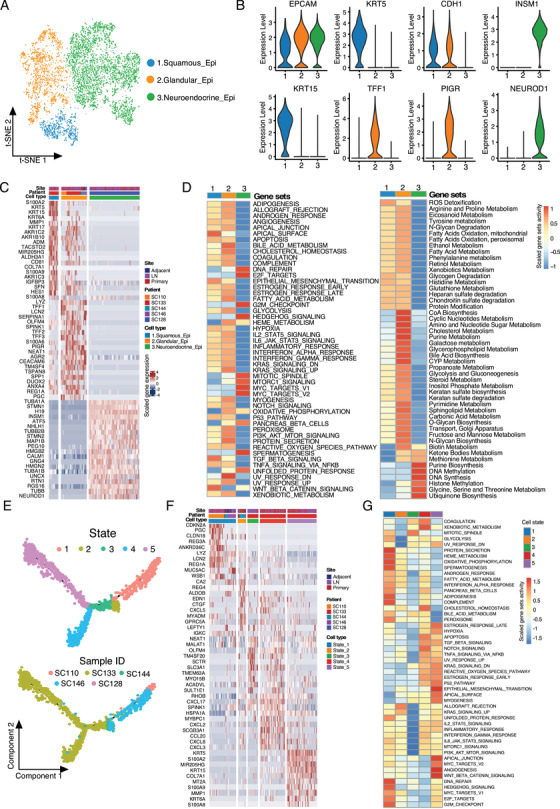
Cellular heterogeneity within the gallbladder epithelial and neuroendocrine tumor cells. (A) The t‐SNE plot of the EPCAM+ cells identified three major cell clusters including squamous, glandular, and neuroendocrine epithelial cells. Resolution used for t‐SNE cell grouping analysis 1.2. (B) Violin plots showing the expression levels of biomarkers in the three major cell clusters identified in A. (C) Heatmap of the top 20 differentially expressed genes (based on Wilcoxon test) for each EPCAM+ cell subgroup. (D) The competitive GSEA analysis of the hallmark gene sets (left) and metabolic activity gene sets (right) between the cell subgroups. (E) Monocle 2 trajectory analysis of the epithelial cells derived from GC and adjacent normal tissues annotated by the cellular state (upper) and patient ID (lower). (F) The top 10 differentially expressed genes (based on Wilcoxon test) between the cell state in (E). (G) The competitive GSEA analysis of the hallmark gene sets between the cell subgroups in (E)

Through integrating the EPCAM+ epithelial cells and NET cells, we identified three distinct cell clusters according to the expression levels of canonical biomarkers (Figures 2A, 2B, 2C, S2A and Table S3). The conventional epithelial cells were CDH1+, whereas CDH1− cells express neuroendocrine biomarkers (INSM1 and NEUROD1).^14^, ^15^ These CDH1−/INSM1+/NEUROD1+ cells were annotated as the NET cells (Figures 2A, 2B, and S3A). Two cell subclusters were noticed in CDH1+ epithelial cells. One cell group with high expression of the keratins including KRT5, KRT15, KRT6A, and KRT17 was annotated as squamous cells, and the other cell group highly expressing the secretary proteins or cytokines including trefoil factors (TFF1, 2, and 3), LCN2, LYZ, and S100A6 was annotated as glandular epithelial cells (Figures 2A, 2B, and S2A). The glandular and squamous epithelial cells were dominantly identified in adenocarcinoma, adenosquamous cell carcinoma tumors, and adjacent normal tissues, whereas the NET cells were exclusively identified in primary and lymph node metastatic NET tissues from NET patient SC128 (Figures S2B–S2E). Competitive gene sets enrichment analysis (GSEA) further revealed the heterogeneity of these epithelial cells (Figure 2D). NET cells showed high activities in proliferation, DNA synthesis, and purine biosynthesis; glandular cells showed stronger activities of bile acid metabolism, cholesterol metabolism, and protein secretion, whereas squamous cells showed reduced activities of these processes (Figure 2D). Due to the different origins of NET and epithelial GC tumor cells, we analyzed the gallbladder epithelial and neuroendocrine cells separately.

For gallbladder adenocarcinoma and adenosquamous cell carcinoma, through transcriptional trajectory analysis, we noticed five different epithelial states from the primary, lymph node metastatic, and adjacent normal tissues (Figure 2E). Among them, states 1 and 2 consisted of nonmalignant glandular epithelial and malignant glandular epithelial in adenocarcinoma; states 3 and 4 were malignant glandular epithelial in adenosquamous carcinoma, whereas state 5 mainly contains squamous epithelial tumor cells in adenosquamous carcinoma (Figures S2F, G, and H). The top DEGs between the cellular states were shown as Figure 2F. Competitive GSEA enriched the bile acid metabolism and protein secretion activities in states 1 and 2, reduced inflammation‐related activities in state 3, and increased angiogenesis and metastasis processes in state 5 (Figure 2G). Given the glandular gallbladder tumor as the most common GC in the clinic, we compared the glandular epithelial between normal and tumor tissues and found that extracellular matrix (ECM)–receptor interaction and focal adhesion processes were enriched from the upregulated genes in tumors, whereas antigen processing and presentation, intestinal immune network for IgA production, and complement and coagulation cascades were enriched from the downregulated genes in tumors (Figure S2I), suggesting the low immunogenicity and responses in GC.

**FIGURE 3:**
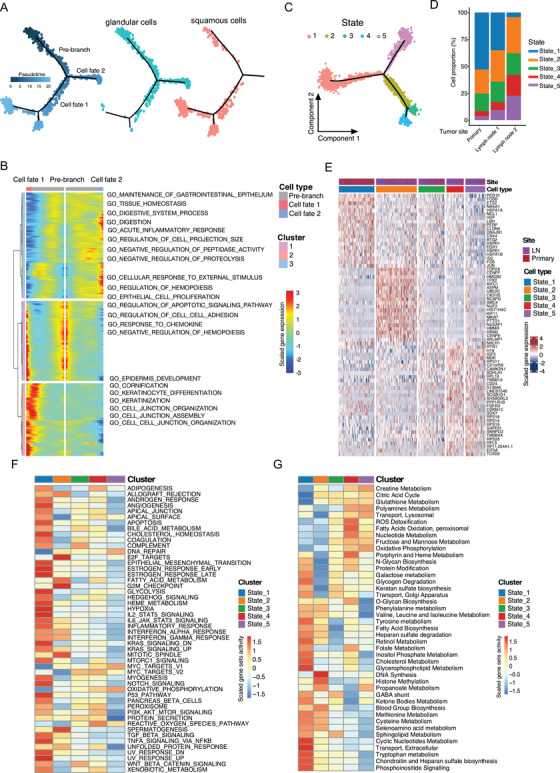
Trajectory analysis of the gallbladder squamous and neuroendocrine tumor cells. A, The branch trajectory plot inferred by Monocle 2 of the epithelial tumor cells derived from adenosquamous cancer patient SC133 (left panel). The distribution of glandular (middle panel) and squamous (right panel) tumor cells in the trajectory was shown. The branch state of the cells in the trajectory plot was indicated by color. (B) The heatmap of differentially expressed genes (in rows, *q*‐value < 10^−10^) along with the pseudotime in each branch, which were hierarchically clustered. The top annotated GO biological processes terms in each cluster were provided. (C) Monocle 2 trajectory analysis of the NET cells derived from patient SC128 annotated by the cellular state. (D) The proportion of the NET cells with distinct states in the tissue origin of SC128 was shown. (E) The top 10 differentially expressed genes (based on Wilcoxon test) between the cell state in (D). (F) The competitive GSEA analysis of the hallmark gene sets between the NET cell subgroups. (G) The competitive GSEA analysis of the metabolic activity gene sets between the NET cell subgroups

In epithelial GC tissues, no apparent EPCAM+ cell composition change was observed between primary and lymph node metastatic tissues (Figure S2C). Interestingly, for the epithelial derived from adenosquamous cell carcinoma (patient SC133), the cells were exclusively enriched with states 3, 4, and 5 (Figures 2D and S2F), covering both glandular and squamous epithelial tumor cells. To infer the relationship between these two cell types, we analyzed the trajectory of tumor cells from the patient SC133 and noticed a branched continuous cell transformation with the squamous epithelial tumor cells at the late stage (cell fate 1) while glandular epithelial at the early stage (cell fate 2) (Figure 3A). The biological processes that involved in the adenocarcinoma to squamous cell carcinoma transformation included the increment of epidermis development, cornification, keratinization activities, and so on, and the reduction of digestion system process, acute inflammatory response, tissue homeostasis, and maintenance of gastrointestinal epithelium according to gene ontology (GO) analysis (Figure 3B).

We also performed the trajectory analysis of NET cells from primary and lymph node metastatic tissues and identified five cell states (Figure 3C), which showed different proportions in primary and lymph node metastatic tumors (Figure 3D). The primary tumor consisted of state 1 dominantly while metastatic lesion contained higher proportions of states 4 and 5 (Figure 3D). Consistently, NET cells in state 1 showed a higher expression of cancer stem cell biomarkers (CD44, VGF, and ID2, etc.) and elevated activities of bile acid‐related metabolism and inflammation, whereas state 2 showed enhanced cell proliferation and downregulated cell junction process (Figures 3E, 3F, and 3G). In states 3, 4, and 5, the cells showed upregulated oxidation‐related processes and fructose and mannose metabolism (Figures 3F and 3G). These results suggest that oxidative‐related activities were enhanced while the immune responses were lost during the lymph node spread of NET cells.

Taken together, these results demonstrated the heterogeneity of malignant cells in GC and the dynamic cell status transition during GC progression.

### 2.3 Distinct subpopulations of TILs in GC

**FIGURE 4:**
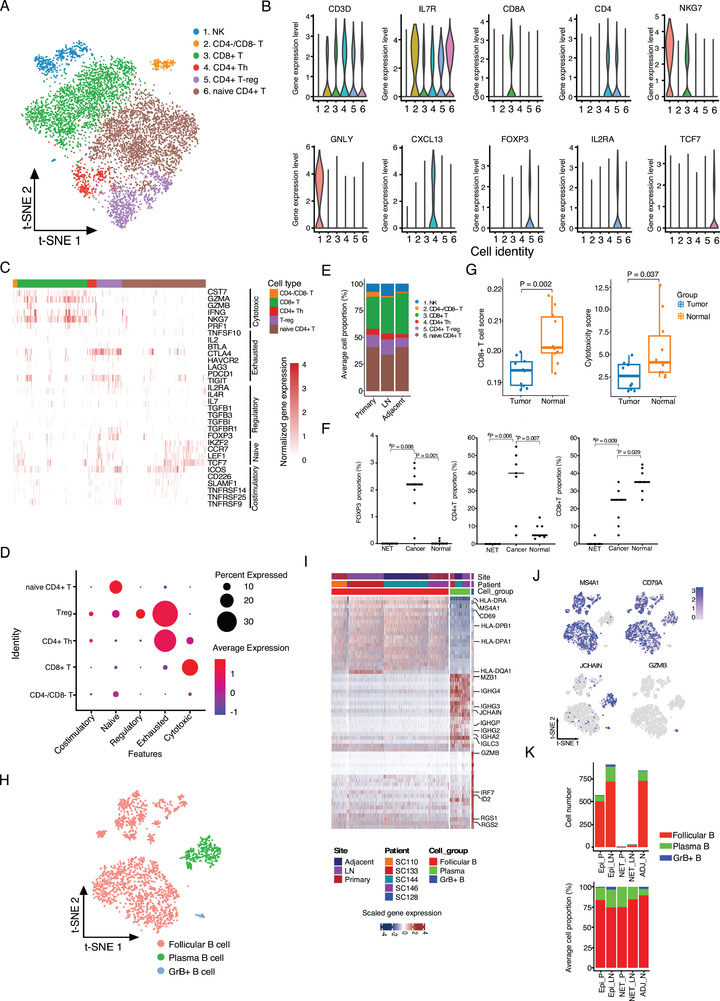
Diversity and functionality of tumor‐infiltrating lymphocytes in GC tissues. (A) The t‐SNE plot of the lymphocytes was shown, colored by the cell subgroups as indicated. Resolution used for t‐SNE cell grouping analysis is 2.0. (B) The violin plot of the normalized expression levels of representative biomarkers in each cell subgroup. (C) The normalized expression profile of biomarkers related to distinct cellular biological activities or T cell state was shown. (D) Dot plots of representative cytotoxic, exhausted, regulatory, naïve, and costimulatory signatures in T cells. *Z*‐score normalized of GSVA enrichment scores. (E) The averaged proportion of lymphocytes in primary tumor (*n* = 4), lymph node metastatic (LN, *n* = 4), and adjacent normal (*n* = 2) tissues was shown, colored by cell subgroups. (F) Comparison of FOXP3+, CD4+, and CD8+ cell proportion in the NET (*n* = 4), glandular tumor (*n* = 7), and the adjacent normal (*n* = 7) tissues. ^*^Paired or ^#^ unpaired Student's *t*‐test was performed. (G) Comparison of the estimated CD8+ T cell proportion and cytotoxicity score signature in 10 paired tumor and normal tissues of GSE139682 database. Two‐tailed paired Student's *t*‐test. (H) The t‐SNE plot of B cells was shown, colored by cell subgroups as indicated. (I) The top 20 differentially expressed genes (based on Wilcoxon test) for each B cell subgroup as indicated. (J) The t‐SNE plots of the B cell biomarkers (MS4A1, CD79A, JCHAIN, and GZMB), colored by the normalized gene expression level in the cells. Resolution used for t‐SNE analysis is 1.0. (K) The total B cell number (left panel) and average cellular proportion (right panel) of distinct subgroups in different tissue types. Epi_P, primary epithelial (adenocarcinoma or squamous) tumor tissue; Epi_LN, lymph node metastatic epithelial (adenocarcinoma or squamous) tumor tissue; NET_P, primary neuroendocrine tumor; NET_LN, lymph node metastatic neuroendocrine tumor; ADJ_N, adjacent normal tissue

T&NK lymphocytes were identified as the most abundant TME cell populations of GC (Figure 1), which were further annotated as six subpopulations (Figures 4A, 4B, S3A–S3D, and Table S4), including NK, CD4‐/CD8‐ T, CD8+ T, CD4+/CXCL13+ T helper (CD4+ Th), CD4+ T‐reg, and naïve CD4+ T cells. Among them, the five subpopulations of T cell showed distinct activities. CD8+ T cells showed strongest cytotoxic signature (Figure 4D) with high levels of cytotoxic‐related genes (CST7, GZMA, GZMB, IFNG, and NKG7); however, a small proportion of CD8+ T cells expressed the immune checkpoint genes including CTLA4, TIGIT, and PDCD1 (Figure 4C), suggesting their exhaustion state. CD4+/CXCL13+ Th and CD4+/FOXP3+ T‐reg cells were enriched as the most exhausted and costimulatory groups, which highly expressed the immune checkpoint genes (CTLA4 and TIGIT) and the costimulatory genes (ICOS, TNFRSF9, TNFRSF14, and TNFSR25) (Figures 4C and 4D). CD4+ T‐reg cells also showed strongest regulatory signature (Figure 4D). These results suggest the inducible and persistence of the immunosuppressive activities of CD4+ T‐reg and CD4+ Th in GC tissues. The naïve CD4+ T cells showed high level of the naïve feature and related markers (CCR7, LEF1, TCF7), and also the costimulatory genes including ICOS, CD226, and SLAMF1 (Figures 4C and 4D); however, a proportion of the naïve CD4+ T cells was negative for these markers (Figure 4C), suggesting a cellular heterogeneity of this subgroup.

We next compared the subclusters of T/NK cells in gallbladder tumors and normal tissues. For both epithelial GC tumors and adjacent normal tissues, naïve CD4+ T and CD8+ T cells were the dominant subclusters (Figures 4E and S3B). Compared with the adjacent normal tissues, the proportions of CD4+ Th and CD4+ T‐reg cells in primary and lymph node metastatic tumors were increased, whereas the level of infiltrated CD8+ T cells was lower (Figure 4E), representing an immunosuppressive TME. To validate the scRNA‐seq results, we analyzed independent gallbladder adenocarcinoma and adjacent normal tissues by immunohistochemistry (IHC) staining (Figures 4F and S3E) and found a significant increase of FOXP3+ or CD4+ cells and a reduction of CD8A+ cells in tumors (Figure 4F). In addition, by immune infiltration analysis, we found the scores of CD8+ T cell infiltration and cytotoxicity were lower in a public dataset (GSE139682) including 10 pairs of GC and normal tissues (Figure 4G),^16^ further confirming our scRNA‐seq results. We also found that high CD8A but not FOXP3 expression in GC tumors was associated with better OS in a cohort of 289 GC patients (Figure S3F). Unlike epithelial GC, we hardly noticed any T/NK lymphocytes in the primary or metastatic NET tissue in SC128 (Figure S3B), which was confirmed by IHC staining of tumors from other NET patients (Figure S3E).

Tumor‐infiltrating B cells are associated with improved OS of cancer patients that received immunotherapy^17^; however, the roles and characteristics of B cells in GC are largely unknown. Through integrating analysis of all B cells, we identified three major B cell clusters from GC patients (Figure 4H), including MS4A1+/CD79A+ follicular B cell, plasma B cells expressing immunoglobulins (IGHG1, IGHG4, IGLC3, and IGHA2 etc.), and granzyme B‐secreting B cells (GrB+ B) (Figures 4I, 4J, and Table S5). Among them, the follicular B cells were observed as the most abundant B cells in GC tissues (Figure 4K), which showed high level of MHC II molecules such as HLA‐DRA, HLA‐DPB1, HLA‐DPA1, and HLA‐DQA (Figure 4I), suggesting a potential antigen‐presenting role of follicular B cells in gallbladder tissues. GrB+ B cells can secrete the cytotoxic protease granzyme B, suggesting that these B cells may involve cellular cytotoxicity activities.^18^ Similar to T&NK cells, B cells were rarely detected in the NET tissues (Figure 4K), which further suggests the poor immune infiltration phenotype.

### 2.4 Distinct subpopulations of myeloid cells in GC tissues

**FIGURE 5:**
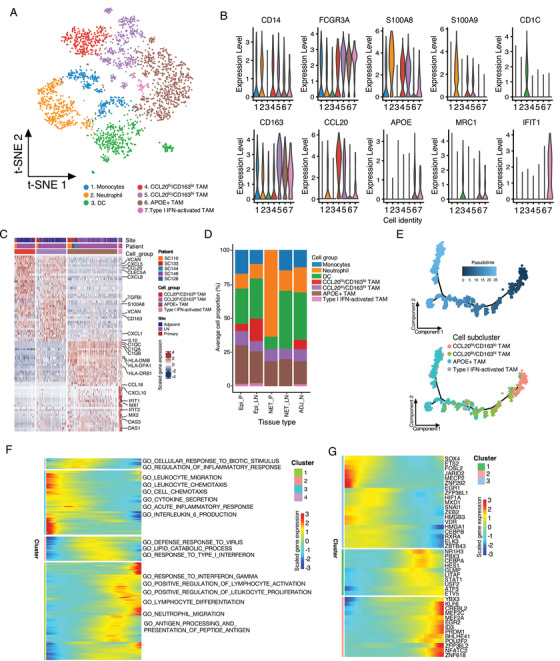
Distinct lineages and functionality of the myeloid cells in GC tissues. (A) The t‐SNE plot of the myeloid cells was shown, colored by the cell subgroups as indicated. Resolution used for t‐SNE cell grouping analysis is 1.0. (B) The violin plot of the normalized expression of representative biomarkers in subclusters was shown. (C) The top differentially expressed genes (based on Wilcoxon test) between the monocyte and macrophage groups. (D) The averaged cellular proportion of the myeloid subgroup cells from different types of tissue was shown (Epi_P, *n* = 4; Epi_LN, *n* = 3; NET_P, *n* = 1; NET_LN, *n* = 1; ADJ_N, *n* = 3). (E) Monocle 2 trajectory analysis of the macrophages annotated by pseudotime (upper panel) and cell subgroups (lower panel) in gallbladder tumor and adjacent normal tissues. (F) The heatmap of differentially expressed genes (in rows, *q*‐value < 10^−10^) along with the pseudotime (annotated in Figure 4D) in the cell trajectory of macrophages were hierarchically clustered into four subclusters. The top annotated GO terms in each cluster were provided (left panel). (G) Heatmap of differentially expressed transcription factors along with the pseudotime in the trajectory analysis of macrophages was shown. Epi_P, primary epithelial (adenocarcinoma or squamous) tumor tissue; Epi_LN, lymph node metastatic epithelial (adenocarcinoma or squamous) tumor tissue; NET_P, primary neuroendocrine tumor; NET_LN, lymph node metastatic neuroendocrine tumor; ADJ_N, adjacent normal tissue

Myeloid cells play critical roles in the antigen‐presentation and inflammation responses. Subclustering of the myeloid cells in GC identified the monocytes, neutrophils, dendritic cells (DCs), and macrophages (Figure 5A). The most abundant myeloid cells, macrophages, were further annotated as four distinct cell groups including CCL20^hi^/CD163^lo^ macrophages, CCL20^lo^/CD163^hi^ macrophages, APOE+ macrophages, and type 1 IFN (T1‐IFN) activating macrophages according to the canonical expression markers (Figures 5B, S4A, and S4B, and Table S6). CCL20^hi^/CD163^lo^ macrophages showed a high expression of proinflammatory cytokines (CCL20, IL1B, and CXCL8) and a low level of M2 macrophage markers (CD163 and MRC1) (Figures 5B, 5C, and Table S6), whereas the expression patterns for CCL20^lo^/CD163^hi^ and APOE+ macrophages were opposite (Figure 5C). APOE+ macrophages also highly expressed the anti‐inflammatory markers including C1QA, C1QB, C1QC, and MSR1 (Figure 5C). In contrast to epithelial GC, the NET tumors harbored only a small number of myeloid cells, mainly neutrophils and DCs (Figures 5D, S4C, and S4D). For epithelial GC, the APOE+ macrophages accounted for the majority of myeloid cells. Interestingly, the anti‐inflammatory macrophages (CCL20^lo^/CD163^hi^ and APOE+) were increased in tumors compared with adjacent normal tissues, whereas the proinflammatory macrophages (CCL20^hi^/CD163^lo^) were reduced (Figures 5D , S4C, and S4D). T1‐IFN‐activated macrophages showed remarkable gene expressions related to type 1 interferon (IFN) response, such as the CXCL10, IFIT1, IFIT2, OAS2, OAS3, MX1, and MX2 (Figure 5C and Table S6).^22^, ^23^

To depict the transition between these macrophages, we performed an unsupervised trajectory analysis and noticed a continuous status transformation from CCL20^hi^/CD163^lo^, CCL20^lo^/CD163^hi^, and T1‐IFN macrophages to APOE+ macrophages in GC (Figures 5E, S4E, and S4F). Along with the cellular trajectory of macrophages, the GO enrichment analysis identified that the processes including response to IFN‐γ, positive regulation of lymphocyte activation, and lymphocyte differentiation were increased, whereas leukocyte chemotaxis, cell chemotaxis, IL‐6 production, and so on were reduced (Figure 5F). For transcriptional factors, the expressions of KLF6, MEF2A, MEF2C, ID3, NFATC2, and ZNF618 were elevated and SOX4, ETS2, MECP2, VDR, and HMGB3 were decreased (Figure 4E), suggesting the key roles of these genes in the anti‐inflammation reprogramming of macrophages.

DCs can also be subclustered (Figure S5 and Table S7). Most of DCs were annotated as conventional CD1c+ DCs (cDC2) (Figure S5D). The others were C1QC+/C1QB+/CD14+ monocyte‐derived DCs (mo‐DCs) with a high level of proliferating genes (TOP2A, PCNA, TYMS, STMN1, and CDK1), CLEC9A+/CADM1+ DCs (cDC1), and CCR7+/LAMP3+ mature DCs, which highly expressed cytokines CCL17 and CCL22 (Figures S5B, S5C, and Table S7) and may involve in T‐regs chemotaxis. These above results revealed the distinct lineages and states of myeloid cells in the TME of GC.

### 2.5 Remodeling of stromal cells with enhanced angiogenesis in GC

**FIGURE 6:**
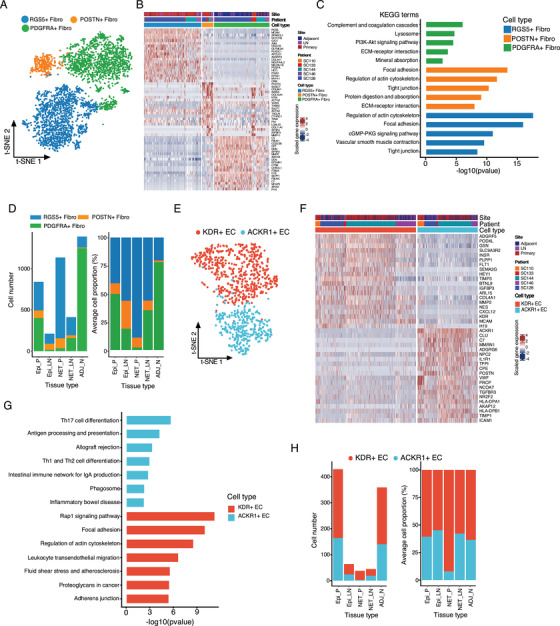
Subclusters analysis of stromal cells in gallbladder tissues. (A) t‐SNE plot of the distinct stromal cell clusters was shown, colored by the identified cell subgroups. Resolution used for t‐SNE cell grouping analysis is 1.0. (B) The top 20 differentially expressed genes (based on Wilcoxon test) between the fibroblast groups. (C) KEGG enrichment analysis of the differentially expressed genes in distinct fibroblast groups. (D) The total cellular number (left panel) and average proportion (right panel) of the three main stromal cells in different tissue types were shown. (E) The t‐SNE plot of endothelial cells was shown, colored by the cell subgroups as indicated. Resolution used for t‐SNE cell grouping analysis is 1.0. (F) The top 20 differentially expressed genes between the two identified endothelial cell subclusters. (G) KEGG enrichment analysis of the differentially expressed genes in in KDR+ or ACKR1+ endothelial cells. (H) The total identified cell number and proportion of different endothelial cells in different tissue types was shown. Epi_P, primary epithelial (adenocarcinoma or squamous) tumor tissue; Epi_LN, lymph node metastatic epithelial (adenocarcinoma or squamous) tumor tissue; NET_P, primary neuroendocrine tumor; NET_LN, lymph node metastatic neuroendocrine tumor; ADJ_N, adjacent normal tissue

Among the 10 cell populations from the whole scRNA‐seq data (Figure 1B), three clusters of stromal cells (RGS5+, PSOTN+, and PDGFRA+ fibroblasts) were further analyzed (Figures 6A, S6, and Table S8). PDGFRA+ fibroblasts were characterized with high expression of well‐known fibroblast markers (COL1A1, DCN, LUM, and PDGFRA) (Figure 6B, S6A, and Table S8), and the notable processes of complement and coagulation cascades, and mineral absorption (Figure 6C), suggesting their roles in the tissue hemostasis of gallbladder. Consistently, PDGFRA+ fibroblasts comprised the main stromal cell types in the adjacent normal tissues (Figures 6D and S6B–S6D). PSOTN+ fibroblasts were characterized with higher expression of extracellular matrix genes including POSTN, TAGLN, FN1, and the α‐smooth muscle actin (ACTA2) gene (Figures 6B, S6A, and Table S8). KEGG enrichment identified high protein digestion and absorption activities (Figure 6C), indicating these cells may contribute to the ECM degradation and microenvironment remodeling in GC. RGS5+ fibroblasts were characterized with high level of RGS5, ACTA2, CD146 (also known as MCAM1), ANGPT2, MYH11, and GJC1 (Figures 6B, S6A, and Table S8),^12^ and the enriched processes including vascular smooth muscle contraction (Figure 6C), suggesting a potential involvement of RGS5+ cells in vascular events. The proportion of PDGFRA+ fibroblasts was significantly reduced in primary and lymph node metastatic tumors, whereas PSOTN+ and RGS5+ cells were significantly increased compared with adjacent normal tissues (Figures 6D and S6A–S6D), reflecting the general remodeling of extracellular stroma in tumor tissues.

**FIGURE 7:**
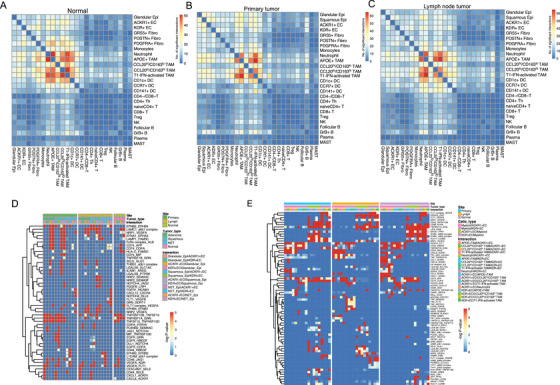
Intercellular ligand–receptor interactions in GC tissues. (A–C) Heatmap depicting the significant ligand–receptor cellular interactions in the normal tissues (A), primary epithelial tumors (B), and lymph node metastatic tissues (C). (D) Heatmap depicting the significant ligand–receptor interactions among gallbladder epithelial malignant/nonmalignant cells, neuroendocrine tumor cells, and endothelial in different tissue types. (E) Heatmap depicting the significant ligand‐receptor interactions between myeloid cells and endothelial in distinct tissue types

Clustering of the endothelial cells identified two clusters termed KDR+ (also known as VEGFR‐2) and ACKR1+ EC cells according to the canonical marker expression (Figures 6E, 6F, and S7A). KDR and Flt‐1 (VEGFR‐1, which was also positive in KDR+ ECs) serve as the receptors for angiogenesis factors VEGFA and VEGFB, indicating that KDR+ ECs were involved in the angiogenesis in gallbladder tissues (Figure S7A).^26^ ACKR1 serves as the atypical receptor for CXC and CC subfamilies including CCL2, CCL5, CCL7, CXCL5, CXCL8, MCP‐1, and so on,^27^ which can sustain the abluminal to luminal transcytosis of tissue‐derived chemokines and their subsequent presentation to circulating leukocytes, thus facilitate the recruitment of myeloid cells and leukocytes in TME of GC.^28^ In comparison, ACKR1+ ECs showed enriched genes involved in the Th17 cell differentiation, antigen processing and presentation, Th1 and Th2 cell differentiation, intestinal immune network for IgA production, and so on (Figure 6G and Table S9), suggesting that ACKR1+ ECs may modulate the T cells differentiation in gallbladder tissues. Both cell types were noticed in GC and normal tissues of individual patients and a slightly increased of ACKR1+ ECs was noticed in the epithelial GC tissues (Figures 6H and S7B–S7D). The above results suggest that stromal cells and endothelial remodeling may enhance angiogenesis in GC.

### 2.6 Intercellular ligand–receptor interactions in GC

To elucidate the molecular interactions between the cell subgroups in GC and adjacent normal tissues, we constructed the origin‐specific cellular communication networks using the CellPhoneDB algorithm^29^ and substantiated the significant interactions between tumor cells, immune cells, and stromal cells. Of them, the ligand–receptor interactions between anti‐inflammatory macrophages (CCL20^lo^/CD163^hi^ macrophages, APOE+ macrophages, and T1‐IFN activating macrophages), neutrophils, and DCs are most significant, followed by the interactions between anti‐inflammatory macrophages and tumor cells/endothelial both in tumor and normal tissues (Figures 6A–6C). Interestingly, unlike anti‐inflammatory macrophages, the proinflammatory macrophages (CCL20^hi^/CD163^lo^ macrophages) showed weak communications with the above cell types (Figures 6A–6C). Cellular interactions between tumor cells and endothelial, especially the CXCL1/ACKR1 and CXCL8/ACKR1 axes which may recruit ACKR1+ ECs, were enhanced in primary tumor tissues (Figures 6A–6D). Compared with adenocarcinoma, squamous cell carcinoma showed strong communications with endothelial by the MIF/TNFRSF10D, EGFR/GRN, EGFR/HBEGF, and EPHB2/EFNB2 interactions (Figures 6B and 6D). For macrophage–epithelial interaction, notably, we noticed significantly increased interactions between ACKR+ ECs and anti‐inflammatory macrophages in primary and lymph node metastatic tissues through LGALS9 in ACKR1+ ECs and its receptors (CD44, SORT1, HAVCR2, and LRP1) in macrophages (Figure 6E), which may contribute to the recruitment and status transition of macrophages in GC.^30^

## 3 DISCUSSION

GC is a malignant disease with a poor prognosis. For the past decades, TME‐targeting strategy provides novel therapeutic choices for cancer treatment; however, as the cellular characteristics and immune microenvironment of GC are largely unknown, these regimes have yet been applied for GC patients in clinic. In this study, for the first time, we depicted the single‐cell transcriptomic profiling of primary gallbladder tumors, lymph node metastatic tumors, and the adjacent normal gallbladder tissues to unveil the cellular heterogeneity of GC and the landscape of subpopulations in TME. The characteristics of gallbladder adenocarcinoma, adenosquamous carcinoma, and NET, as well as their differences, were elaborated. Adenocarcinoma cells lost the antigen‐presenting MHC II molecules and can transdifferentiate to the squamous tumor cells. The immunosuppressive TME in epithelial GC consist of infiltrating CD4+ T‐reg, CXCL13+ Th cells, CCL20^lo^/CD163^hi^, and APOE+ macrophages; however, it is the immune‐desert phenotype for gallbladder NET. Remodeling the stromal and endothelial cells in the GC tissues promotes the angiogenesis and lymphangiogenesis to sustain the growth and metastasis of GC. These results resolve single‐cell transcriptomic profiling of GC tissues, improve our current understanding of GC development and progression, and provide novel therapeutic targets for GC in the future.

Gallbladder adenocarcinoma and adenosquamous carcinoma are originated from the epithelial cells in the gallbladder, whereas the NETs in gallbladder derive from the neuroendocrine cells or peptidergic neural crest Kulchitsky cells.^13^ In the current study, three distinct subpopulations of EPCAM+ epithelial tumor cells were clustered including adenocarcinoma‐derived glandular cells, squamous cell carcinoma, and neuroendocrine carcinoma cells. Each subpopulation was enriched with distinct biological processes or signaling pathways, displaying loss of function as the normal gallbladder epithelial and acquisition of other properties in terms of growth, metabolism, or disease, especially for subpopulations in squamous cell carcinoma and NET cells. The intratumoral heterogeneity and diverse properties of tumor cells make it difficult to exert a broad and effective therapeutic effect for GC through mono‐target drugs or treatments. Interestingly, in the adenosquamous GC patient (SC133), there is a coexistence of squamous and glandular epithelial tumor cells, which showed distinct gene profiling. We noticed a continuous transformation and the shared dominant CNVs of squamous and glandular malignant cells in patient SC133, indicating that squamous cell carcinoma could be differentiated from a subcluster of adenocarcinoma cells in GC, which is consistent with the previous report that the glandular cells could transdifferentiate into squamous cells.^31^ Along with the transdifferentiation, cornification and keratinization were increased, whereas bile acid and fatty acid metabolism activities were lost. More studies are warranted to elucidate environmental and intrinsic factors for cellular transdifferentiation.

The single‐cell portrait of GC microenvironment also supports that immunotherapy could be potentially effective for GC patients. Abundant and diverse types of lymphocytes, including NK cells and multiple subtypes of T cells, were identified in gallbladder adenocarcinoma, suggesting a highly immune infiltrated “hot tumor” phenotype, which usually indicates a good response to immunotherapy.^32^ For CD8+ T cells, although the cytotoxic molecules (GZMA, GZMB, NKG7, and PRF1) were highly expressed, a fraction of CD8+ T cells showed positive with exhaustion biomarkers (CTLA4, PDCD‐1, and TIGIT), indicating their tumor‐cytotoxic activity was constrained. Among CD4+ T cells, naïve CD4+ T, CXCL13+/CD4+ Th, and CD4+ T‐reg cells showed anti‐immunological activity and predominantly inhabited in tumor tissues, suggesting an immunosuppressive environment. Notably, the immune checkpoint proteins, CTLA‐4 and TIGIT, were highly expressed in T‐reg cells, suggesting that CTLA‐4 and TIGIT blockade, may exert effective therapeutic effects for GC treatment. Similar to lymphocytes, multiple subclusters of myeloid cells were noticed in the TME of gallbladder adenocarcinoma tissues, with anti‐inflammatory macrophages as the dominant cell populations. We noticed a serial transformation of macrophages from proinflammatory to anti‐inflammatory status in GC tissues. Also, the anti‐inflammatory rather proinflammatory macrophages elevated interaction with other cells in GC, suggesting the key roles of macrophages in the immunosuppressive microenvironment formation, which may serve as a potential therapeutic target in GC treatment.

Angiogenesis and lymphangiogenesis are crucial in GC carcinogenesis and progression. In our study, we found that the RGS5+ fibroblasts that involved vascular‐related processes were significantly increased in GC tissues compared with adjacent normal tissues. Combined with the finding of enrichment of KDR+ ECs from endothelial cells, these results reflect that enhanced angiogenesis may account for the major event of tissue remodeling during the GC progression. Another enriched epithelial cluster is ACKR1+ ECs, which are involved in leukocyte cell–cell adhesion, leukocyte adhesion to vascular endothelial cell, and leukocyte migration,^34^ thus may reflect the lymphangiogenesis in tumor tissues. These results are consistent with the high immune infiltration we found. In addition to mediating the infiltration of lymphocytes, the interaction between these stromal cells and lymphocyte/myeloid cells may also tune the inflammatory status of tumor‐infiltrating cells, which merits further exploration. In addition, ECs could function as the semi‐professional antigen‐presenting cells and trigger T‐cell costimulation and specific immune‐cell activation.^35^ In gallbladder tissues, only ACKR1+ ECs (rather than KDR+ ECs) showed positive expression of MHC II molecules and high activities of Th1, Th2, and Th17 cell differentiation, suggesting these ECs may involve immune surveillance and mediate the T cells differentiation

Gallbladder NET is a rare type of gallbladder tumor, which is different from conventional epithelial gallbladder adenocarcinoma. The clinical manifestation, treatment, and prognosis of gallbladder NET are rarely reported. Through single‐cell transcriptomic profiling, we identified novel biomarkers for gallbladder NET cells and discovered multiple differences of gallbladder NET from gallbladder adenocarcinoma. First, the CNV score of gallbladder NET cells was not elevated both in primary and lymph metastatic tumors, suggesting a small genetic variation, at least at copy number levels. Second, gallbladder NET cells exhibited distinct clusters with other gallbladder tumors, which is consistent with the distinct tumor origin. Third, unlike adenocarcinoma as a “hot tumor,” gallbladder NET tumors harbored rare immune cells. The “cold tumor” or immune desert microenvironment as well as the small genetic variation implies that gallbladder NET may not respond well to immunotherapy and tumor cell‐targeted therapy may be an efficient therapeutic strategy. However, due to the limitation of clinical samples, this hypothesis should be verified with more studies.

In summary, we resolved the single‐cell atlas of human GC and the microenvironment. Our comprehensive characterization of cell subtypes from normal, primary, and lymph node metastatic tumor tissues reveals the cellular heterogeneity and differential lineages and uncovers the immunosuppressive environment and subpopulations in GC development and progression. These findings provide deep insights into the GC characteristics and potential therapeutic targets in the future.

## 4 METHODS

### 4.1 Patients recruitment for scRNA‐seq analysis

Three gallbladder adenocarcinoma patients, one gallbladder adenosquamous cell carcinoma patient, and one gallbladder neuroendocrinology tumor (NET) patient who received the curative surgery treatment in Zhongshan Hospital affiliated to Fudan University were recruited from March 2019 to July 2019. All patients have been histologically confirmed by the hematoxylin–eosin staining method, and they had not yet received any pretreatment of the diseases. Of them, four are female and one is male, with the age range between 52 and 82 years old. Detailed clinical characteristics of individual patients that included in the scRNA‐seq analysis was provided in Table S1. The written consent of each patient has been provided, and the study was approved by the Institutional Review Board of Fudan University Zhongshan Hospital.

### 4.2 Single‐cell sample preparation

After the routine surgery treatment, the primary, lymph node metastatic tumor samples and adjacent normal tissues of the gallbladder were freshly collected, cut into pieces (2–4 mm in size) with the sterile scissors on ice. The tissues were dissociated with the Solo™ Tumor Dissociation Kit (#JZ‐SC‐58201; Sinotech Genomics, Shanghai, China) at 37°C for 60 min. The tissue pieces were mixed by pipetting up and down every 20 min. After stopping digestion with excess DMEM medium, the cells were filtered with a 70 μm nylon cell strainer and centrifuged to remove the enzymes in a 15 ml centrifuge tube at 800 × *g* for 5 min. The red blood cells were removed with red blood cell lysis buffer (#420301; BioLegend, San Diego, CA, USA). Then the cells were washed twice with PBS buffer, and resuspended in 3 ml of DMEM medium. The cellular viability was checked by 0.4% Trypan blue stain.

### 4.3 Single‐cell library preparation and sequencing

The microbeads‐captured single‐cell library construction was performed with BD Rhapsody™ Single‐Cell Analysis System (#633701; BD Biosciences, CA, USA) following the manufacture's guidelines. To remove the batch effects, the cells from the same patient but with different types of tissues (primary, lymph node metastatic, and/or adjacent normal tissues) were firstly stained using Calcein AM (#C1430; Thermo Fisher Scientific, USA) and Draq 7 (#564904; BD Biosciences) to determine cell number and viability via BD Rhapsody™ Scanner. The single cells from different tissues were multiplex labeled with a unique 45‐nucleotide barcode tag using BD Human Single‐Cell Multiplexing Kit that based on the antibody‐oligo technology (#633781; BD Biosciences) following the manufacture's guidelines.^36^ These tag‐labeled cells were equally pooled together and randomly loaded in one BD Rhapsody™ Cartridge containing more than 200,000 microwells. Then, the cell capture beads labeled with unique molecular identifiers (UMI) were added onto the cartridge excessively to ensure that nearly each microwell contains one bead. After washing away the excess beads, the lysis buffer was added to lyse the cells that allow the hybridization of RNA molecules with the beads. Beads were harvested into a single tube where the double‐stranded cDNA was synthesized through several steps including reverse transcription, second strand synthesis, end preparation, adapter ligation, and transcriptome amplification. Then, the final cDNA library was generated with double strands full‐length cDNA by random priming amplification. The cDNA library was generated with BD Rhapsody™ WTA Reagent Kit (#633802; BD Biosciences). Meanwhile, the SampleTag library was generated from microbeads‐captured single‐cell SampleTag sequences through several steps including reverse transcription, nest PCR and final index PCR using the BD Rhapsody™ WTA Reagent Kit (#633802; BD Biosciences). All constructed libraries were sequenced on a NovaSeq instrument (Illumina) using the PE150 mode (Pair‐End for 150 bp read).

### 4.4 Single‐cell sequencing data processing

The sequencing data were analyzed with the standard BD Rhapsody™ Whole Transcriptome Assay Analysis Pipeline on Seven Bridges (https://www.sevenbridges.com) according to the manufacture's recommendations, which included filtering by reads quality, annotating reads, annotating molecules, determining putative cells, and generating single‐cell expression matrix. Briefly, the FASTQ files generated from the NovaSeq were filtered to remove reads with low sequencing quality (reads length < 64 bases for R2 and base quality score < 20). The R1 reads were annotated to get the cell label index, UMI information, and poly‐dT tail sequence. In reads annotation step, R2 reads were mapped to Genome Reference Consortium Human Build 38 (GRCh38) using STAR (version 2.5.2b). In molecules annotation step, the recursive substitution error correction and distribution‐based error correction (DBEC) algorithms were used to adjust artifact molecules due to amplification bias. The DBEC‐corrected reads counts were used to calculate the minimum second derivative along with the cumulative reads and a filtering algorithm was applied to identify the putative cells. For cellular tag assignment, the cells with a minimum read count > 75% of total reads were defined as a singlet, the cells with the count for two more tags > 20% were labeled as multiplets, otherwise, the cells were recognized as undetermined. Only singlets were included for further analysis. UMI count per cell corrected by the DBEC algorithm was applied to generate the single‐cell expression matrix for each sample.

### 4.5 Cell subtypes identification using the t‐SNE method

The output of the cell‐gene count matrix was processed with the Seurat (v 3.1.0) package of R software (version 3.6.1) for quality control and down‐streaming analysis.^37^ Low‐quality cells with <200 genes or with >40% mitochondrial genes were removed from the analysis. As the cells from tumor and adjacent normal tissues were loaded in batch for each patient, the data for each patient as individual Seurat Object. The Seurat object for each patient was integrated with the harmony algorithm (R package, Harmony, version 1.0).^38^ The top 50 principal components (PCAs) were used for graph‐based clustering to identify a distinct group of cells at the indicated resolution. In the subgroup analysis, significant PCAs identified with the ElbowPlot() function were used for graph‐based clustering for each cell cluster to identify subgroup cells based on the t‐SNE analysis.^37^ The cell types of the identified cells were defined based on their expression of the canonical marker genes: epithelial cells (EPCAM, CDH1, KRT17, and KRT19), neuroendocrine cells (EPCAM, INSM1, and NHLH1), B cells (MS4A1, CD79A, JCHAIN, and CD19), T cells (IL7R, CD3D, and GZMB), NK cells (NKG7), myeloid cells (CD74, CD14, and LYZ), RGS5+ fibroblasts (RGS5, ACTA2, LUM, and DCN), PSOTN+ fibroblasts (ACTA2, COL1A1, and LUM), PDGFRA+ fibroblasts (LUM, DCN, COL1A1, and PDGFRA), endothelial cells (vWF and PECAM1), and mast cells (CPA3 and MS4A2).

### 4.6 DEGs and GSEA

The specific markers for each clusters were identified though conducting the FindAllMarkers function in Seurat package (only.pos = T, min.pct = 0.25) to the normalized expression data.^37^ The genes with adjusted P‐value < 0.05 were considered statistical significance, which were used for KEGG and GO enrichment analysis. The ClusterProfiler package (version 3.14.3) was applied for the enrichment analysis for the cluster‐specific biomarker genes.^39^ GSEA was performed with MSigDB gene sets to identify the differential pathways. GSEA was conducted with modifications reported by Cillo et al.^40^ that was implemented in the SingleSeqGset package (version 0.1.2), which applied variance inflated Wilcoxon rank‐sum testing to determine the gene sets enrichment across the cell clusters.^40^ The 50 hallmark gene sets in the MSigDB databases (https://www.gsea‐msigdb.org/gsea/msigdb) and curated metabolic activity gene sets derived from Qian et al.^41^ were used for the competitive GSEA analysis. The full gene lists of T cells signature (including the cytotoxic, exhausted, regulatory, naïve, and costimulatory activity of T cells) were extracted from the published report by Chung et al.^42^

### 4.7 CNV score inference

The gallbladder epithelial cells in tumor tissues may contain the malignant tumor cells as well as the residual nonmalignant cells. To separate the nonmalignant population from the definitive tumor cells, we evaluated the genetic aberrations by CNV score, which inferred from the RNA sequencing data of single cells similar to the methods reported by Kim et al.^12^ In brief, we first applied the inferCNV algorithm (version 1.2.2) implemented in R package (https://github.com/broadinstitute/inferCNV/wiki) for each tumor sample to infer the CNV scores of each gene locus within individual chromosomes with parameters set as denoise = TRUE, analysis_mode = “samples,” window_length = 101, cutoff = 0.1, and max_centered_threshold = 3.^43^ Then, we summarized the CNV information of signal cells using two parameters: the mean squares of CNV scores and the correlation of the CNV score across all gene locus of each cell with the average CNV score of the top 5% cells with highest mean squares CNV scores. The epithelial cells showing CNV signal perturbation (>0.02 mean squares of CNV estimates or >0.2 for CNV correlation with top 5% CNV score cells) were classified as malignant cells. The malignant cell identification was only applied for the glandular and squamous epithelial tumor cells but not for NET cells as no significant CNV change was noticed for NET cells. Comparison of CNV scores between the NET or glandular with the adjacent normal glandular cells were performed using the two‐tailed Wilcoxon tests.

### 4.8 Inference of tumor cell state and immune cells development using the trajectory analysis

The malignant cell clusters from the scRNA‐seq data of GC patients were extracted at first. The single‐cell trajectory was created with the Monocle (version 2.18.0) based on the UMI counts of the selected cells.^44^ The newCellDataSet() function of Moncole2 and parameter expressionFamily = negbinomial.size() were applied to create an object. The dispersionTable() function of Monocle 2 was used to select genes for trajectory inference and calculate a smooth function to describe expression variance across cells along with the mean expression level, and only genes with mean expression ≥ 0.1 were used for the analysis. The variable genes among the cells selected by Seurat were subjected to dimension reduction through reduceDimension() function and parermeters reduction_method = “DDRTree” and max_components = 2. The single cells were in further ordered and visualized through the plot_cell_trajectory() function of Monocle 2 to infer the trajectory of the cells. The genes with expression levels changed along with the pseudotime of trajectory were calculated (*q*‐val < 10^−10^). The pseudotime‐dependent genes were divided into subgroups according to their gene expression patterns and visualized with the plot_pseudotime_heatmap() of Monocle 2. The GO terms in each cluster were evaluated with the clusterProfilter package (version 3.18.0).

To infer the developmental trajectory of the TAM cells, we first selected the CCL20^hi^/CD63^lo^ macrophages, CCL20^lo^/CD163^hi^ macrophages, type I IFN activating macrophages, and APOE+ macrophages clusters and the UMI counts of these cells were used as an input to Monocle 2. Similar to the tumor epithelial cells, the newCellDataSet() function was applied to create a Monocle 2 object. And the variable genes among the cells selected by Seurat were subjected to dimension reduction. The TAMs were ordered and visualized through plot_cell_trajectory() heatmap of Monocle 2. The genes that changed along with pseudotime of the development trajectory were calculated (*q*‐val < 10^−10^) and visualized with plot_pseudotime_heatmap() of Monocle 2. These pseudotime‐dependent genes were clustered into subgroups according to their gene expression patterns and the GO terms analysis was performed using clusterProfilter package (version 3.18.0).

### 4.9 GC patient cohort and immunohistochemistry staining of immune genes

To determine the prognosis values of CD8A+ T cells and FOXP3 Treg cells infiltration level in GC patients, the formalin‐fixed paraffin‐embedded (FFPE) GC tissues from 289 patients were recruited. Detailed information of most patients has been reported in previous study.^8^ In brief, 289 consecutive patients that received the radical resection or palliative surgery were enrolled from Fudan University Zhongshan Hospital between 2004 and 2013. Clinicopathological information of the patients were collected from the medical records of the patients, and the patients were followed every 3 months in the first 2 years after surgery treatment and every 6 months in the subsequent years. OS time was defined as the day of operation to the date of death or the last follow up (as census status). The FFPE tumor tissues were collected to establish the tissue microarrays (TMA, core diameter, 2.0‐mm). The IHC staining was performed following the general protocols. Briefly, the TMAs were deparaffinized with the xylenes, and rehydrated with gradient ethanol solutions. The endogenous peroxidase activities were blocked with 3% H_2_O_2_ for 30 min with menthol. The antigen retrieval was performed with the heat repair process at 98°C for 45 min with the citrate buffer at pH = 6.0. After three washes with PBS, the slides were incubated with antibodies and diaminobenzidine staining. Then, the sections were stained with hematoxylin. In the final, the slides were dehydrated with gradient ethanol solutions and mounted with neutral balsam before microscopic observation. Specific antibodies used were anti‐FOXP3 (ab22510, 1:100; Abcam, USA) and anti‐CD8 (IR623, 1:100; Dako, Glostrup, Denmark). The immune cells intensity was determined as the proportion of total number of positive markers per field (magnitude, 200×). The patients were categorized into higher or lower group according to the mean value of the immune cell intensity score, and comparison of the OS between groups were performed using Kaplan–Meier plot together with the log‐rank test.

To compare the tissue infiltrated T‐reg, total CD4+ T and CD8+ T cells in NET, adenocarcinoma, and normal gallbladder tissues, another seven paired adenocarcinoma and normal tissues and four NET tissues were enrolled at the Zhongshan Hospital. Similar to above protocols, the IHC staining method was applied to determine the immune cells intensity. The following antibodies in this study were used: anti‐FOXP3 (mouse, 1:200, ab20034; abcam), CD4 (Rabbit, 1:200, ab183685; abcam), and CD8 (Rabbit, 1:200, ab237709; abcam). Comparison of the immune cell intensity was performed using the paired or unpaired Student's *t*‐test (two‐tailed) as indicated.

### 4.10 Correlation to public datasets

RNA‐sequencing study of the gallbladder tumor tissues and the adjacent normal tissues in 10 patients was conducted by Xu et al.^45^ and the processed RPKM (reads per kilobase of transcript per million reads mapped) data were downloaded from the GEO database (accession code: GSE139682). The ROTS (Reproducibility Optimized Test Statistic) pipeline was applied to identify the DEGs between the tumor and normal tissues.^46^ The immune infiltrates analysis based on gene expression in bulk tissues was performed by TIMER2.0 (http://timer.cistrome.org) ^16^. To calculate the T/NK cells‐mediated cytotoxicity score, the mean expression of genes including GZMK, GZMA, GZMB, GZMH, NKG7, PRF1, and GNLY was calculated in the normal and tumor gallbladder tissues of GSE139682. Comparison of the CD8+ T cells infiltration levels and cytotoxicity score was performed using the paired Student's *t*‐test (two‐tailed).

### 4.11 Cell–cell interaction network analysis

The ligand–receptor interactions between the epithelial cells, the endothelial cells, macrophages, and endothelial cells from the primary, lymph node metastatic, and the adjacent normal tissues were mapped using the CellPhoneDB algorithm (www.cellphonedb.org).^29^ This method determined the potential cellular ligand–receptor interactions between the cell clusters based on the gene expression level. The significance of the cellular interactions was calculated based on the 1000 times permutation test. In the current study, we performed the cellular interactions for the ligands and receptors expressed in at least 25% of the cell subsets. We excluded the cellular interactions within the identical cell clusters, the interactions between the collagens and between the cell subsets account for less than 0.1% of the total cells. Those ligand–receptor interactions with *p* < 0.05 from the permutation tests were considered statistically significant.

## CONFLICT OF INTEREST

The authors declare no conflict of interest.

## AUTHOR CONTRIBUTIONS

Peizhan Chen, Qian Ba, Yueqi Wang, Houbao Liu, and Hui Wang conceived and designed the overall study. Yueqi Wang, Xiaobo Bo, Jie Wang, Lingxi Nan, and Changcheng Wang provided samples and clinical annotation and reviewed the clinical data. Peizhan Chen, Jingquan Li, and Qian Ba coordinated the data acquisition and analysis. Peizhan Chen, Qian Ba, Houbao Liu, and Hui Wang interpreted the data. Peizhan Chen, Qian Ba, Yueqi Wang, Houbao Liu, and Hui Wang wrote the manuscript. All authors reviewed and approved the final manuscript.

## Supporting information

Supporting Information

Supporting Information

Supporting Information

Supporting Information

Supporting Information

Supporting Information

Supporting Information

Supporting Information

Supporting Information

Supporting Information

## Data Availability

The data reported in this study are available in the CNGB Nucleotide Sequence Archive (CNSA: https://db.cngb.org/cnsa; accession number: CNP0001599). Specific code will be available upon reasonable request.
