# Supplementary material for: Diversity and intratumoral heterogeneity in human gallbladder cancer progression revealed by single‐cell RNA sequencing
Source: Clin Transl Med. 2021 Jun 27;11(6):e462. doi: 10.1002/ctm2.462 (PMC8236117; doi:10.1002/ctm2.462)
Supplement: Supplementary file 1 — Supporting Information [file CTM2-11-e462-s009.docx]

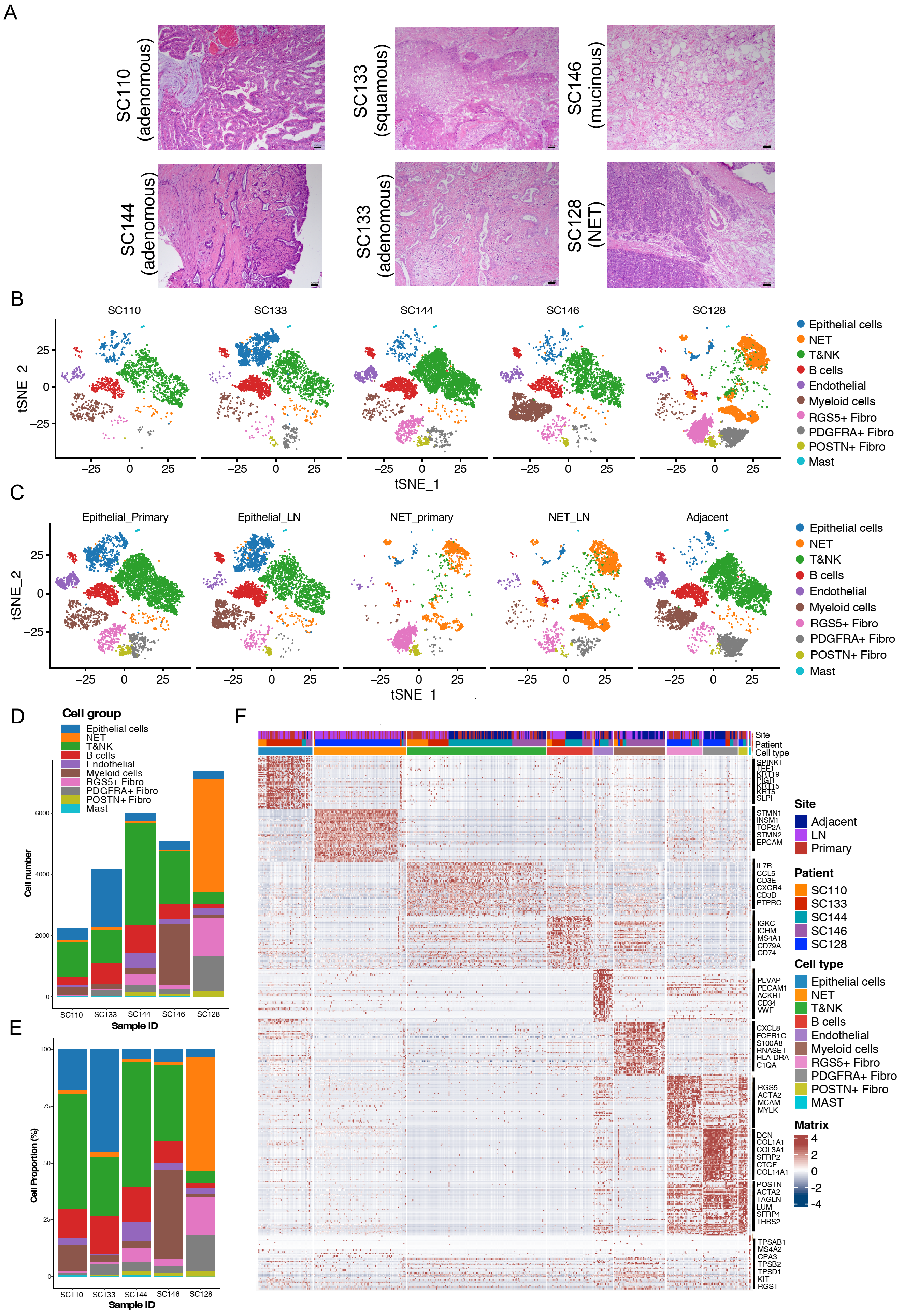
**Figure S1. Distribution of cell subgroups in different patients and tissue types.** A,H&E staining of the tumor tissues from the 5 recruited patients in current scRNA-seq study. B and C, The stratified t-SNE plot of the 10 main cell subgroups according to the individual patient (B) or tissue type (C). D and E, the totally identified cell number (D) and average proportion (E) of the 10 distinct cell subgroups in each gallbladder cancer patients, colored by the cellular types. F, The heatmap of the differentially expressed genes between the cellular groups in gallbladder tissues. The well-known biomarker genes of cellular subgroups were labeled. Epithelial_Primary, primary epithelial (adenocarcinoma or squamous) tumor tissue; Epithelial_LN, lymph node metastatic epithelial (adenocarcinoma or squamous) tumor tissue; NET_Primary, primary neuroendocrine tumor; NET_LN, lymph node metastatic neuroendocrine tumor; Adjacent, adjacent normal tissue.


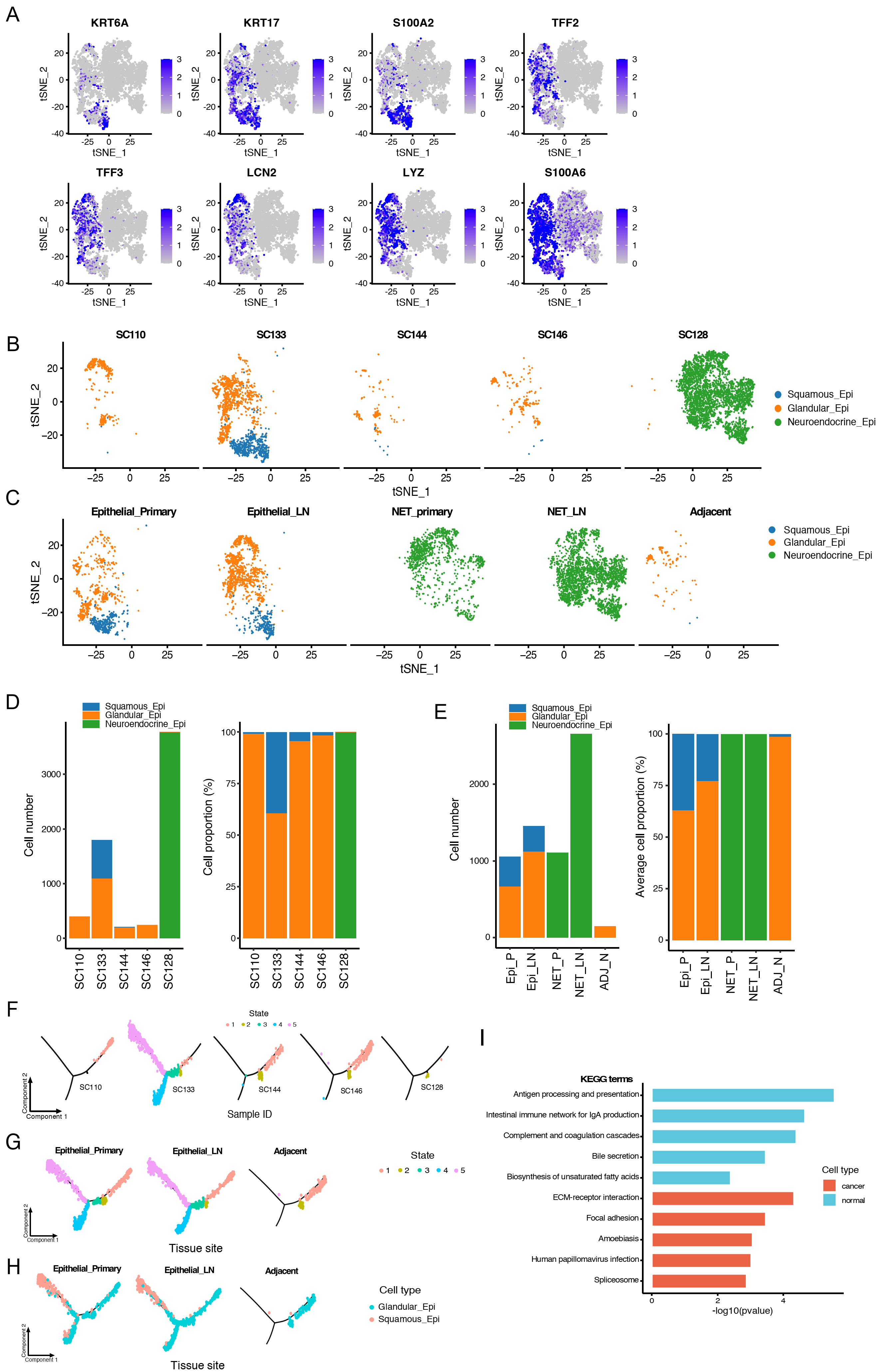


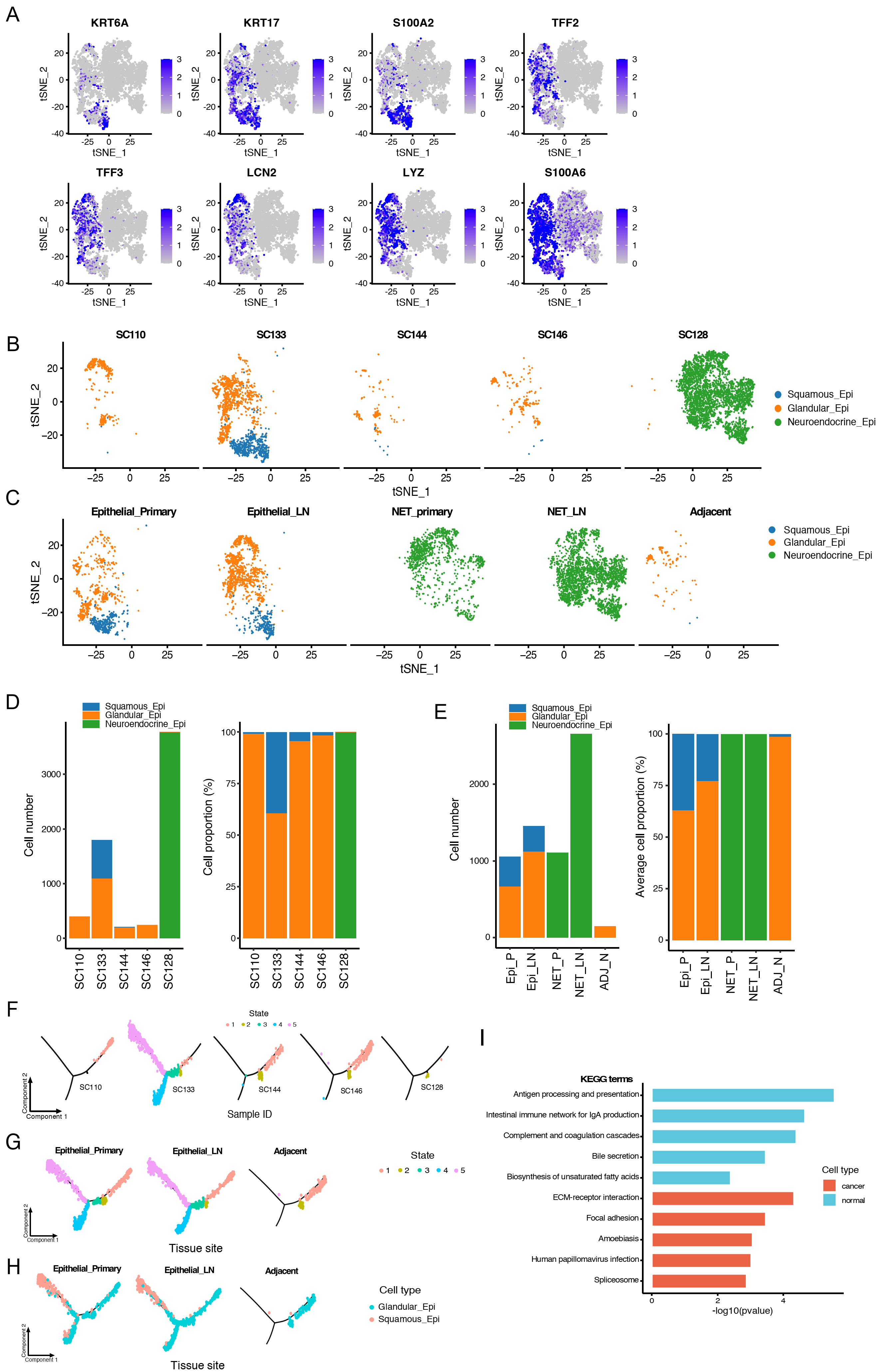
**Figure S2.** **Diversity of the total EPCAM+ cells identified in gallbladder tumor and adjacent normal tissues.** A, Expression levels of representative feature markers of the three EPCAM+ subclusters in gallbladder tissues. Grey to blue color bar indicating ln(normalized count + 1) of gene expression level. B and C, The t-SNE plot of the 3 main EPCAM+ cell subgroups from the individual patient (B) and tissue type (C), colored by the indicated cell types. Diversity of the identified EPCAM+ cells in gallbladder tissues. D and E, The total identified EPCAM+ epithelial cell number and its proportion according to distinct patient (D) or tissue type (E). F and G, The Monocle 2 trajectory analysis of the epithelial cells derived from gallbladder epithelial tumor and adjacent normal tissues stratified by patient (F) and tissue type (G) and colored by the cell states. H, The Monocle 2 trajectory analysis of the epithelial cells stratified by the tissue type and colored by the epithelial types. I, KEGG enrichment analysis of the differentially expressed genes in the normal and tumor glandular cells. Epithelial_Primary (Epi_P), primary epithelial (adenocarcinoma or squamous) tumor tissue; Epithelial_LN (Epi_LN), lymph node metastatic epithelial (adenocarcinoma or squamous) tumor tissue; NET_Primary (NET_P), primary neuroendocrine tumor; NET_LN, lymph node metastatic neuroendocrine tumor; Adjacent (ADJ_N), adjacent normal tissue.


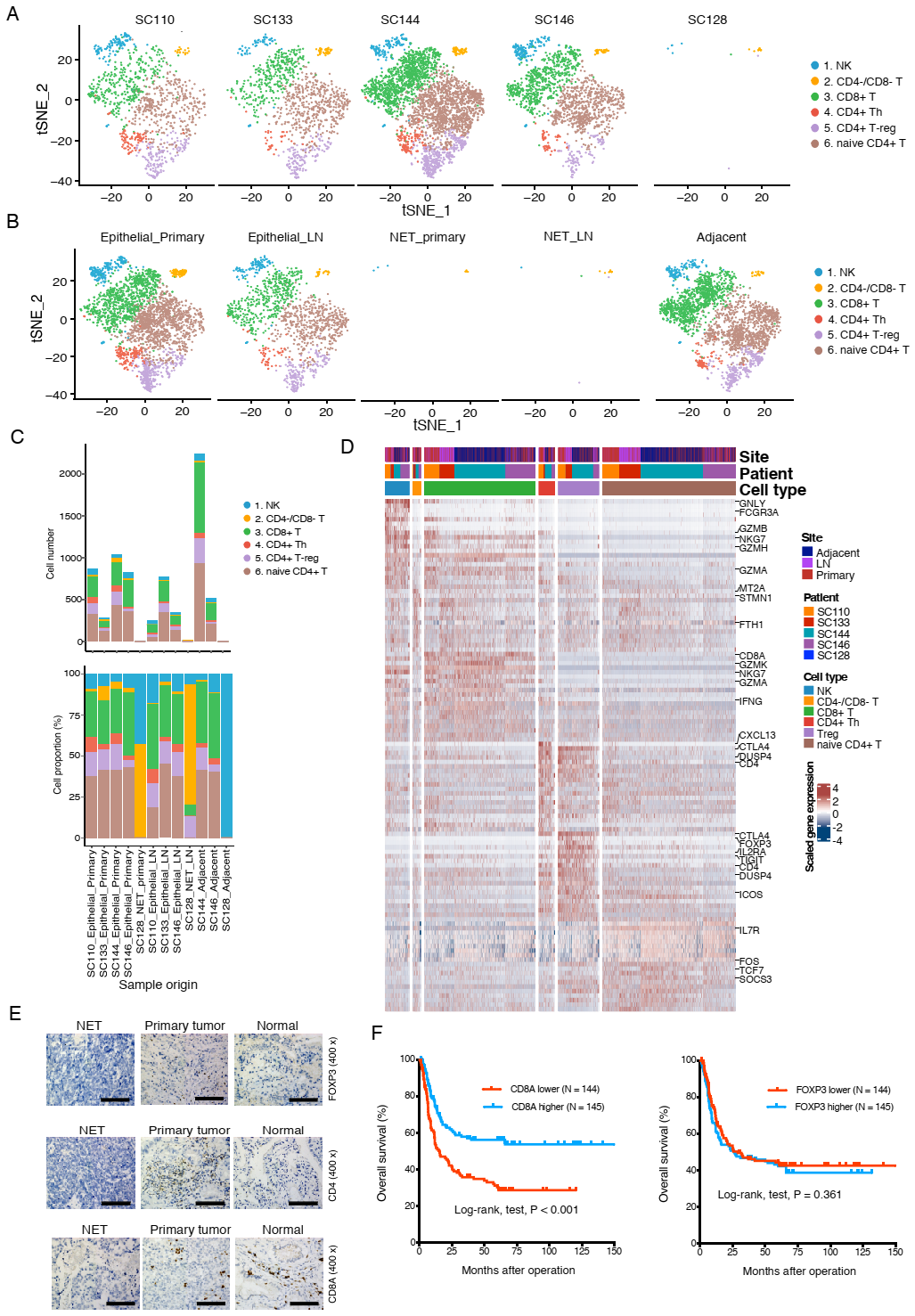


**Figure S3. Distinct subcluster of the lymphocytes in gallbladder tumor and adjacent normal tissues.** A and B, The t-SNE plot of T&NK lymphocytes stratified by the patient (A) and tissue site (B) identified in the current scRNA-seq study. C, The total cell number (upper panel) and proportion (lower panel) of lymphocytes identified from different tissue origins. D, The top 20 differentially expressed genes in distinct T&NK cell clusters. E, Representative IHC staining of the FOXP3, CD4, and CD8A in the normal, NET, and adenocarcinoma tumor tissues. Scale Bar,100 μm. F, The Kaplan-Meier plot of the overall survival for 289 gallbladder cancer patients according to CD8A and FOXP3 expression level (higher vs. lower). Overall survival rate comparison between the groups were performed with Log-rank test.


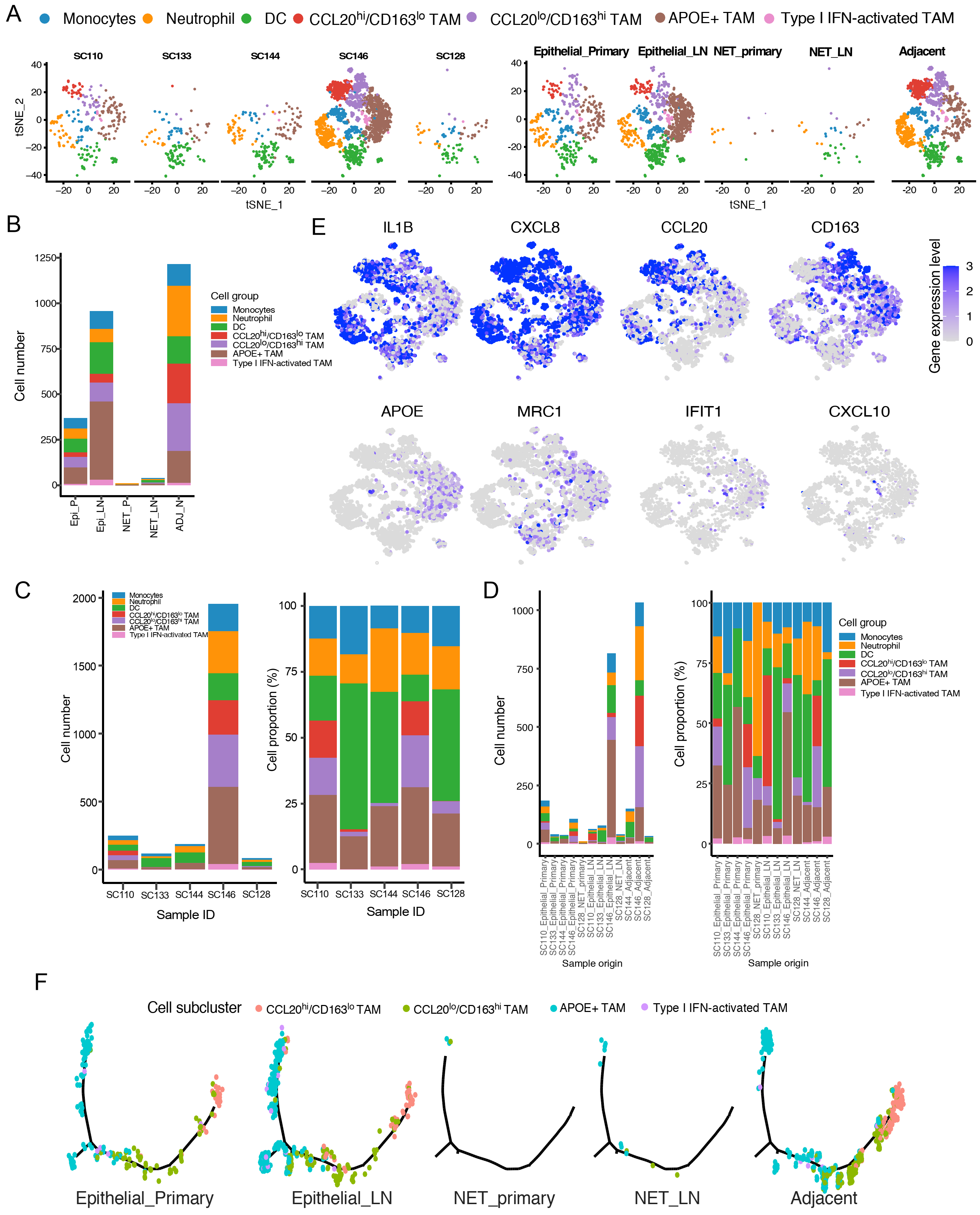


**Figure S4. Distinct subcluster myeloid cells in gallbladder tumor and adjacent normal tissues.** A, The t-SNE plot of myeloid cells stratified by the individual patient (left) and the tissue site (right). B, The total cell number of identified myeloid cells in different tissue origins. C, The total cell number (left panel) and proportion (right panel) of myeloid cells identified in different patients. D, The total cell number (left panel) and proportion (right panel) of myeloid cells identified in different tissue origins. E, Feature t-SNE plots of the myeloid cell biomarkers including the pro-inflammatory genes (IL1B, CXCL8, CCL20), anti-inflammatory genes (APOE, CD163 and MRC1), and the type 1 IFN activated genes (CXCL10 and IFIT1). Color key from grey to blue indicates the normalized gene expression levels of the cells. F, Monocle 2 trajectory analysis of the macrophages stratified by the tissue site. Epithelial_Primary (Epi_P), primary epithelial (adenocarcinoma or squamous) tumor tissue; Epithelial_LN (Epi_LN), lymph node metastatic epithelial (adenocarcinoma or squamous) tumor tissue; NET_Primary (NET_P), primary neuroendocrine tumor; NET_LN, lymph node metastatic neuroendocrine tumor; Adjacent (ADJ_N), adjacent normal tissue.


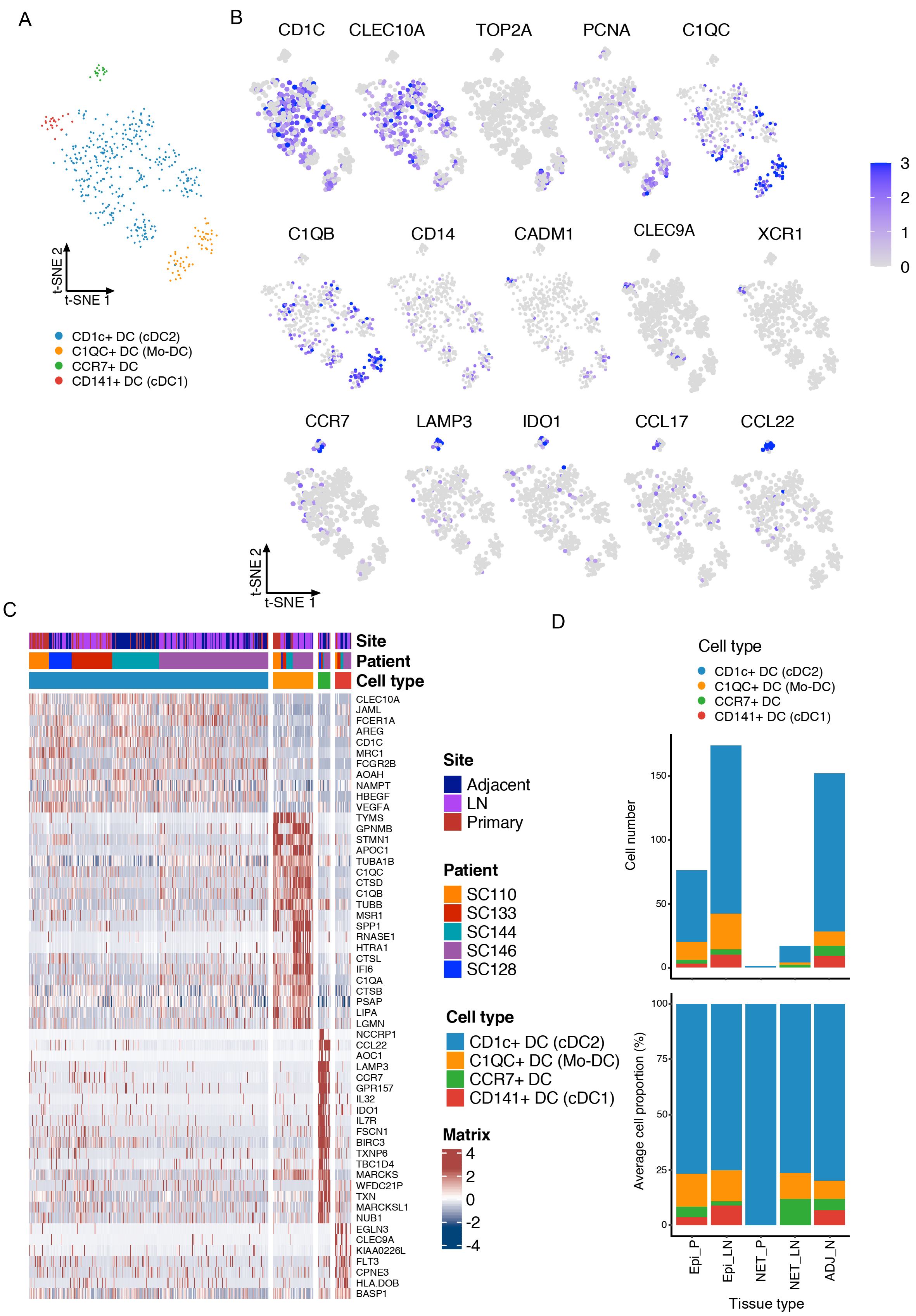


**Figure S5. Distinct subcluster of dendritic cells in gallbladder tissues.** A, The t-SNE plot of dendritic cells in gallbladder tumor and adjacent normal tissues, colored by cell subclusters. B, Feature t-SNE plots of the dendritic cell biomarkers as indicated. Color key from grey to blue indicates the normalized gene expression levels of the cells. C, The differentially expressed genes between the 4 subtypes of dendritic cells (based on the Wilcoxon test). D, The total cell number (upper panel) and average proportion (lower panel) of dendritic cell subtypes identified in different tissue origins. Epithelial_Primary (Epi_P), primary epithelial (adenocarcinoma or squamous) tumor tissue; Epithelial_LN (Epi_LN), lymph node metastatic epithelial (adenocarcinoma or squamous) tumor tissue; NET_Primary (NET_P), primary neuroendocrine tumor; NET_LN, lymph node metastatic neuroendocrine tumor; Adjacent (ADJ_N), adjacent normal tissue.


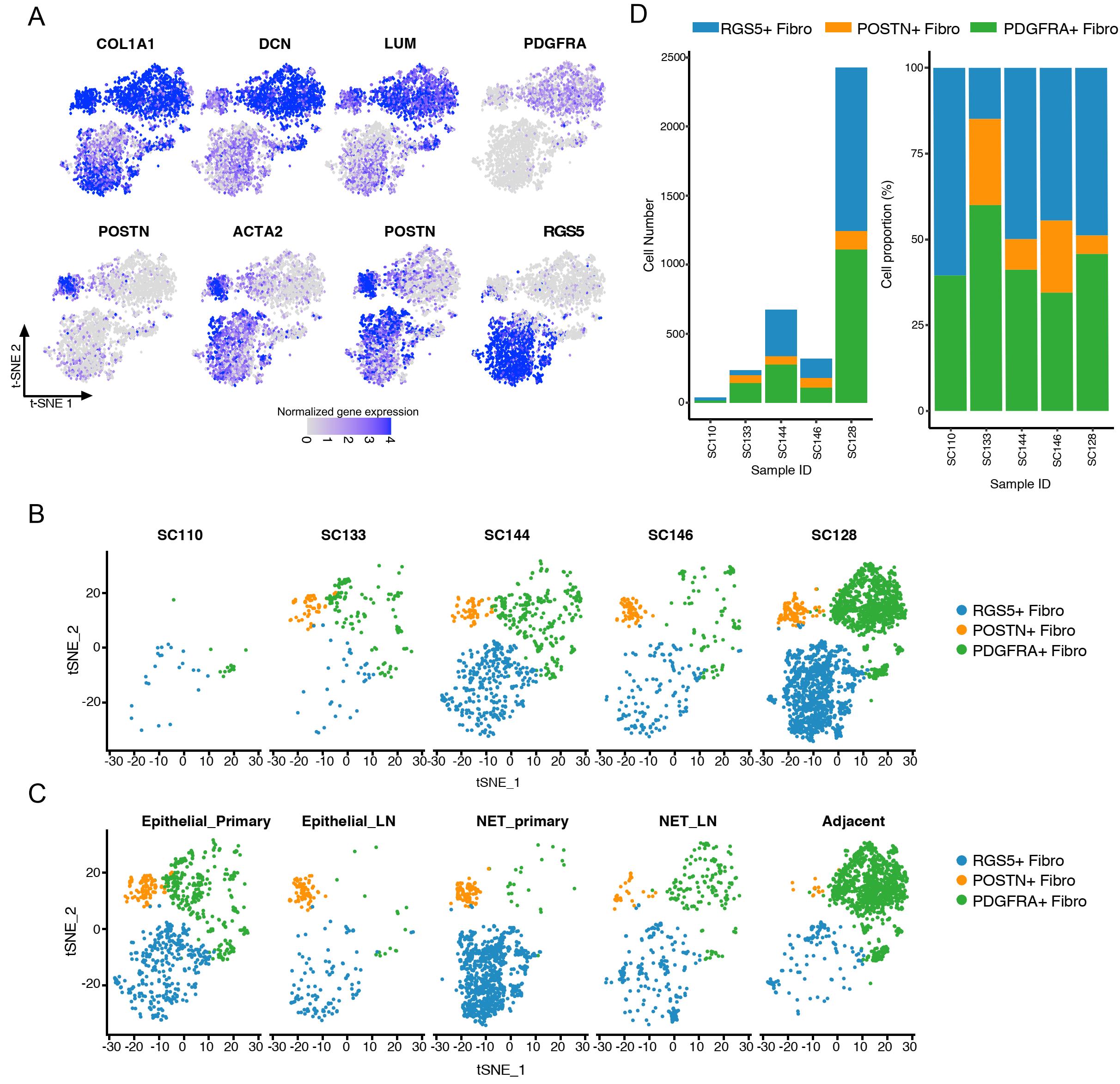


**Figure S6. Distinct subgroups of the fibroblasts in gallbladder tumor and normal tissues.** A, Feature t-SNE plots of the fibroblasts biomarkers as indicated, and colored by the normalized gene expression levels of the cells. B and C, Stratified t-SNE plots of the fibroblasts by patients (B) and tissue sites (C), colored by distinct cell groups. D, The total cell number (left panel) and proportion (right panel) of the 3 main fibroblasts identified in individual patient.


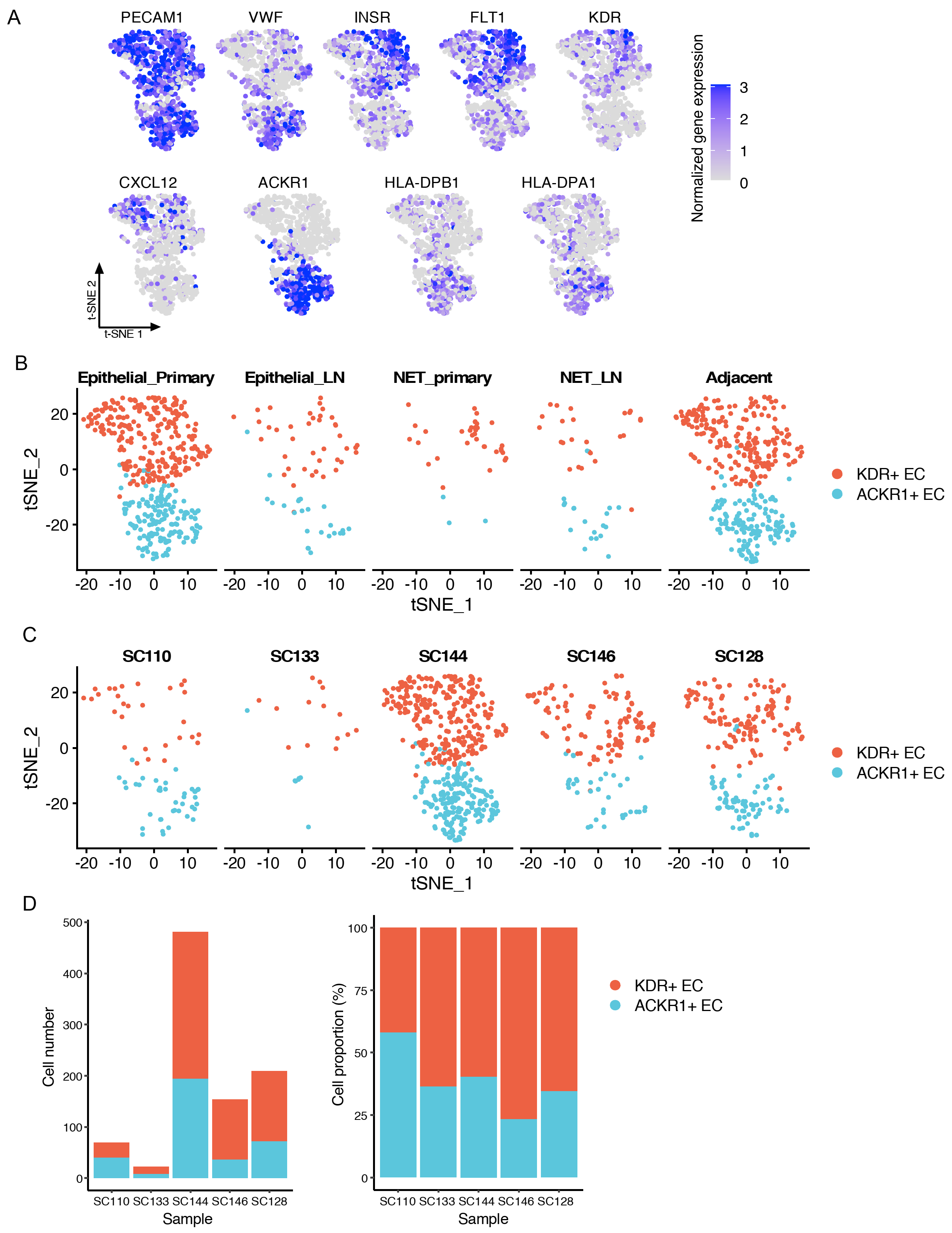


**Figure S7. Distinct subgroups of the endothelial cells in gallbladder tumor and normal tissues.** A, Feature t-SNE plots of the endothelial biomarkers including PECAM, vWF, INSR, FLT1, KDR, CXCL12, ACKR1, HLA-DPB1 and HLA-DPA1, and colored by the normalized gene expression levels of the cells. B and C, Stratified t-SNE plots of the endothelial cells by tissue types (B) and patients (C), colored by distinct cell groups. D, The total cell number (left panel) and proportion (right panel) of the 2 endothelial cells identified in individual patient.
