# Supplementary material for: Diversity and intratumoral heterogeneity in human gallbladder cancer progression revealed by single‐cell RNA sequencing
Source: Clin Transl Med. 2021 Jun 27;11(6):e462. doi: 10.1002/ctm2.462 (PMC8236117; doi:10.1002/ctm2.462)
Supplement: Supplementary file 2 — Supporting Information [file CTM2-11-e462-s002.docx]

**Table S1.** The clinical characteristics of the patients recruited in the scRNA-seq analysis

**Abbreviations:** MSI, microsatellite instability; MSS, microsatellite stable; PD-L1, Programmed death ligand 1.

| **Patient ID** | **Gender** | **Age** | **Tumor subtype** | **Extent of the tumor** | **Nearby lymph node spread** | **Liver invasion** | **Microsatellite Stability** | **PD-L1 expression** | **Samples collection** | **Cell number** | **Mean mitochondrial genes proportion** |
| --- | --- | --- | --- | --- | --- | --- | --- | --- | --- | --- | --- |
| SC110 | Female | 66 | Adenocarcinoma | Serosa | Positive | Negative | MSI | PD-L1 positive | Primary & Lymph node | 2,237 | 18.30% |
| SC128 | Female | 52 | Neuroendocrine carcinoma | Serosa | Positive | Negative | MSS | PD-L1 negative | Primary & Lymph node & Adjacent | 7,381 | 22.26% |
| SC133 | Female | 82 | Adenosquamous carcinomas | Serosa | Positive | Positive | MSS | PD-L1 negative | Primary & Lymph node | 4,165 | 22.20% |
| SC144 | Male | 82 | Mucinous adenocarcinoma | Perimuscular fibrous tissue | Negative | Negative | MSS | PD-L1 negative | Primary & Adjacent | 6,009 | 21.45% |
| SC146 | Female | 79 | Adenocarcinoma | Lamina propria | Positive | Negative | MSS | PD-L1 negative | Primary & Lymph node & Adjacent | 5,095 | 19.13% |
